# Effect of TiO_2_ Morphology on the Properties and Photocatalytic Activity of g-C_3_N_4_/TiO_2_ Nanocomposites Under Visible-Light Illumination

**DOI:** 10.3390/molecules30030460

**Published:** 2025-01-21

**Authors:** Matevž Roškarič, Gregor Žerjav, Janez Zavašnik, Matjaž Finšgar, Albin Pintar

**Affiliations:** 1 Department of Inorganic Chemistry and Technology, National Institute of Chemistry, Hajdrihova ulica 19, SI-1001 Ljubljana, Slovenia; matevz.roskaric@ki.si (M.R.); albin.pintar@ki.si (A.P.); 2 Gaseous Electronics, Jožef Stefan Institute, Jamova cesta 39, SI-1000 Ljubljana, Slovenia; janez.zavasnik@ijs.si; 3 Faculty of Chemistry and Chemical Engineering, University of Maribor, Smetanova ulica 17, SI-2000 Maribor, Slovenia; matjaz.finsgar@um.si

**Keywords:** visible-light photocatalysis, g-C_3_N_4_/TiO_2_, TiO_2_ morphology, dual mixed type-II/Z-scheme, photocatalytic degradation of bisphenol A

## Abstract

This study focused on the preparation and investigation of g-C_3_N_4_/TiO_2_ photocatalysts using different TiO_2_ morphologies (anatase nanoparticles (TPs), poorly crystalline nanotubes (aTTs), and well-crystalline anatase nanorods (TRs)) and self-synthesized g-C_3_N_4_ (CN). The synthesis of the g-C_3_N_4_/TiO_2_ composites was carried out using a mortar mixing technique and a g-C_3_N_4_ to TiO_2_ weight ratio of 1:1. In addition, the g-C_3_N_4_/TiO_2_ composites were annealed in a muffle furnace at 350 °C for 2 h in air. The successful formation of a g-C_3_N_4_/TiO_2_ composite with a mesoporous structure was confirmed using the results of XRD, N2 physisorption, and FTIR analyses, while the results of microscopic analysis techniques confirmed the preservation of TiO_2_ morphology in all g-C_3_N_4_/TiO_2_ composites investigated. UV-Vis DR measurements showed that the investigated g-C_3_N_4_/TiO_2_ composites exhibited visible-light absorption due to the presence of CN. The results of solid-state photoluminescence and electrochemical impedance spectroscopy showed that the composites exhibited a lower charge recombination compared to pure TiO_2_ and CN. For example, the charge transfer resistance (RCT) of the CNTR/2 composite of TR and CN calcined in air for 2 h was significantly reduced to 0.4 MΩ, compared to 0.9 MΩ for pure TR and 1.0 MΩ for pure CN. The CNTR/2 composite showed the highest photocatalytic performance of the materials tested, achieving 30.3% degradation and 25.4% mineralization of bisphenol A (BPA) dissolved in water under visible-light illumination. In comparison, the pure TiO_2_ and CN components achieved significantly lower BPA degradation rates (between 2.4 and 11.4%) and mineralization levels (between 0.6 and 7.8%). This was due to (i) the presence of Ti^3+^ and O-vacancies in the TR, (ii) enhanced heterojunction formation, and (iii) charge transfer dynamics enabled by a dual mixed type-II/Z scheme mechanism.

## 1. Introduction

In heterogeneous photocatalysis, titanium dioxide (TiO_2_) is still of great interest due to its high stability, low cost, unique optical properties, and high photocatalytic activity [1,2,3]. However, its application is limited due to the required excitation using UV light (band gap value 3.0–3.4 eV) and the high tendency for recombination of the generated charge carriers [1,4,5,6]. There are several approaches to improve these drawbacks, including (i) the deposition of plasmonic metals (Au, Pt, etc.) on the surface, (ii) doping or self-doping (Ti^3+^) of TiO_2_, (iii) using different TiO_2_ polymorphs, (iv) forming a heterojunction with a suitable low band-gap semiconductor, etc. [6,7,8,9,10,11,12,13,14,15,16,17,18]. The latter is particularly interesting as it improves the disadvantages of TiO_2_ by using low-cost semiconductors instead of noble metals such as Au. A suitable candidate for forming a heterojunction with TiO_2_ is graphitic carbon nitride (g-C_3_N_4_) [4,18]. g-C_3_N_4_ is a polymeric 2D material with a moderate bandgap (~2.7 eV), high stability, and non-toxicity [4,18,19,20,21,22]. On the other hand, g-C_3_N_4_ typically has a low specific surface area and a high tendency to recombine charge carriers [4,11,19,23,24], suggesting the combination of TiO_2_ with g-C_3_N_4_ to overcome the disadvantages of both semiconductors [18,25,26].

Various complex synthesis methods are used to prepare g-C_3_N_4_/TiO_2_ composites, in which several factors such as the temperature of hydrothermal synthesis, the ratio of components, the microwave power, the duration of synthesis, etc., significantly affect the properties of the obtained materials [27,28,29]. In our previous study, the optimum weight ratio between g-C_3_N_4_ and TiO_2_ was found to be 1:1 [17]. Žerjav et al. [9] showed that the morphology of TiO_2_ can play a crucial role in the photocatalytic activity of TiO_2_-based composites. Jo et al. [30] investigated the influence of TiO_2_ morphology on the activity of g-C_3_N_4_/TiO_2_ composites for isoniazid degradation and found that the enhanced activity of the composites was due to the improved charge separation according to the Z-scheme. They also found that the optimal amount of g-C_3_N_4_ was different when using TiO_2_ nanoparticles (5% g-C_3_N_4_) or nanotubes (3% g-C_3_N_4_). The overall most active composite was the combination of 3% g-C_3_N_4_ and TiO_2_ nanotubes due to the increased carrier mobility and larger specific surface area of the TiO_2_ nanotubes. A search in the literature shows that the exact impact of TiO_2_ morphology (different TiO_2_ morphologies have different surfaces (crystal planes), textures, and optoelectronic properties) on the photocatalytic activity of g-C_3_N_4_/TiO_2_ composites has not yet been fully explored. The novelty of this research lies in the systematic investigation of how the morphology of TiO_2_—in particular nanoparticles, nanotubes, and single crystalline anatase nanorods—influences the formation and properties of the heterojunction between TiO_2_ and g-C_3_N_4_, and how these structural differences affect photocatalytic efficiency. This approach directly addresses gaps in the existing literature by focusing on the role of TiO_2_ morphology in optimizing the performance of g-C_3_N_4_/TiO_2_ composites for improved charge separation and utilization. We prepared g-C_3_N_4_/TiO_2_ composites using commercial anatase TiO_2_ nanoparticles and two internally synthesized (alkaline hydrothermal synthesis) TiO_2_ powders: poorly crystalline nanotubes and single crystalline anatase nanorods. The g-C_3_N_4_/TiO_2_ composites were synthesized using a mortar mixing technique and a 1:1 ratio of g-C_3_N_4_ to TiO_2_. In addition, the g-C_3_N_4_/TiO_2_ composites were annealed in a muffle furnace at 350 °C. An important benchmark for evaluating the efficiency of photocatalytic systems is the photooxidation rate, i.e., the rate at which oxidation reactions occur on the surface of a photocatalyst when it is exposed to light. Several factors influence this rate, including the band gap, crystallinity, morphology, surface area, and charge separation efficiency of the photocatalyst. External conditions, such as light intensity, wavelength, pH, and temperature, also play an important role. Therefore, the synthesized composites were characterized using surface and chemical-sensitive analysis techniques (EPR, XPS, FTIR, UV-Vis DR, and TEM). In addition, the investigated g-C_3_N_4_/TiO_2_ composite photocatalysts were analyzed for possible wastewater remediation under visible-light illumination, using the endocrine disrupting compound bisphenol A (BPA) dissolved in water as a model organic pollutant. By using coumarin and DMPO/DMSO as probe molecules in conjunction with in situ BPA quenching experiments, we were able to gain an insight into the generation and utilization of the reactive oxygen species and determine the possible reason for the different photocatalytic behavior of the investigated g-C_3_N_4_/TiO_2_ composites.

## 2. Results and Discussion

### 2.1. Structural and Chemical Analyses

#### 2.1.1. FTIR Analysis

**Figure 1:**
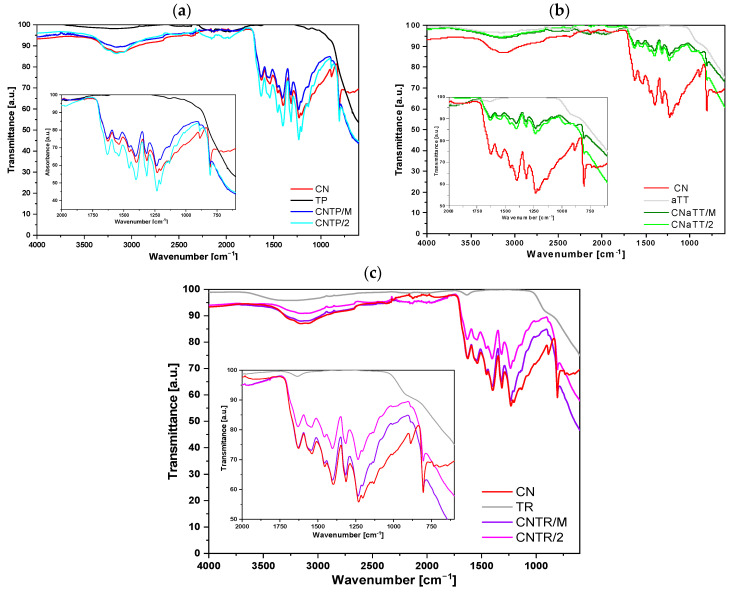
ATR-FTIR spectra of the investigated nanosolids recorded at room temperature. Figure (**a**) shows the TP series, (**b**) shows the aTT series and (**c**) shows the TR series. The insets show the characteristic vibrations of g-C_3_N_4_.

To confirm the successful synthesis of CN and its incorporation into the composite photocatalysts, we performed FTIR measurements; the results are shown in Figure 1a–c. It can be observed that pure CN exhibits characteristic vibrations for g-C_3_N_4_ (1100–1700 cm^−1^) and the characteristic breathing mode of the triazine or tri-s-triazine structural unit (heptazine) at 808 cm^−1^ [31,32,33,34]. In particular, the FTIR bands at 1206, 1234, and 1314 cm^−1^ originate from the C–NH–C unit, while the signals in the range between 1600 and 1350 cm^−1^ correspond to vibrations of the condensed C–N–C structural units, with the bands at 1315 cm^−1^ belonging to C–N stretching and at 1629 cm^−1^ to C=N stretching [31,32,33,34]. The broad peak centered at 3100 cm^−1^ originates either from water adsorbed on the surface and/or from NH_x_ functional groups left over from CN synthesis. For all pure TiO_2_ components, we can observe the characteristic Ti-O-Ti stretching (600–1100 cm^−1^) in addition to the presence of surface adsorbed water and/or -OH functional groups (peak at ~3100 cm^−1^). The characteristic peaks of g-C_3_N_4_ and TiO_2_ are present in all investigated photocatalysts, which suggests the successful synthesis of a heterojunction between g-C_3_N_4_ and TiO_2_ independent of the TiO_2_ morphology used. No other additional peaks were observed, confirming that the applied synthesis approach did not alter the chemical composition of the two components and did not introduce any impurities. From the results of the elemental analysis (Table S1) of the fresh samples, it can be concluded that the pure TiO_2_ samples contained small amounts of carbon originating from the air impurities adsorbed on the surface. The CN solid had the expected carbon and nitrogen content and a small amount of hydrogen from the non-condensed NH_x_ functional groups. All composite photocatalysts showed an increased carbon and nitrogen content, demonstrating the successful addition of CN with TiO_2_. Furthermore, the carbon and nitrogen content were similar in all composites regardless of the TiO_2_ used, which means that they all contained a similar amount of g-C_3_N_4_. Therefore, possible changes in photocatalytic behavior were due to the intrinsic changes of the hybrids and were not influenced by the content of the CN component. Additional annealing of the mortar composites slightly reduced the amount of carbon and nitrogen, as a small amount of CN was removed from the samples during this procedure. However, the CN content did not change significantly.

#### 2.1.2. XRD Analysis

**Figure 2:**
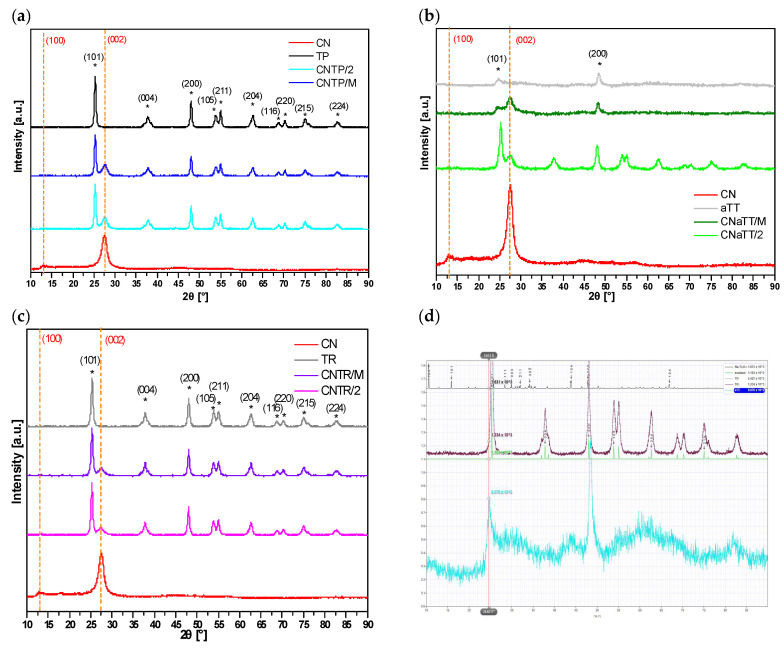
XRD patterns of the investigated photocatalysts (**a**–**c**) and Na-titanate simulation (**d**). The orange dashed lines represent the characteristic peaks of g-C_3_N_4_ (PDF ICDD 00-066-0813) and the * the characteristic peaks of anatase TiO_2_ (PDF ICDD 01-086-1157).

The phase composition and purity of the investigated materials were determined using XRD analysis. From the XRD diffractograms in Figure 2a–c, it can be seen that both TiO_2_ and g-C_3_N_4_ components are present in all composite photocatalysts. For the CN component, the characteristic peak of g-C_3_N_4_ corresponding to ICDD 00-066-0813 can be observed at about 27.5° 2θ for the (002) plane in all cases [30], while the weakly expressed (100) peak at 13.2° 2θ is not visible. In all samples containing TiO_2_, we can observe characteristic peaks corresponding to the TiO_2_ anatase polymorph (PDF ICDD 01-086-1157). However, for the pure aTT and CNaTT/M samples, only two characteristic peaks can be observed (anatase (101) at ~25° 2θ and anatase (200) at ~48° 2θ). Before calcination, the particles were either poorly crystalline or the crystallites were very small (~10 nm), resulting in the typical diffuse peaks with low intensity. When the CNaTT/M composite was annealed at 350 °C for 2 h, the crystallinity improved and more characteristic TiO_2_ anatase peaks are visible in the diffractogram of the CNaTT/2 sample; even the low temperature of 350 °C was sufficient to transform the poorly crystalline TiO_2_ into well-crystalline TiO_2_. The shape of the TiO_2_ peaks and the crystallite size of TiO_2_ remained the same or slightly increased in the case of the CNTP/M composite (Table 1). Using the simulation of Na-titanate in Figure 2d, we confirmed the absence of any sodium contamination in our aTT or TR containing samples. This proves, also considering the results of the FTIR measurements, that both components were present in the composites without significantly changing their chemical or phase composition. However, a slight shift in the g-C_3_N_4_ (002) peak can be observed in all composites compared to pure CN (Table 1), suggesting the successful formation of a heterojunction, as the separation of the layers probably changes due to the interactions with TiO_2_ [35].

#### 2.1.3. Determination of Porosity of Samples

Nitrogen adsorption/desorption experiments were carried out to obtain information on the porosity of the tested samples. All photocatalysts show isotherms (Figure S1a) that correlate with the IUPAC nomenclature of type IV(a) with an H3 hysteresis loop, indicating a mesoporous structure with slit-like pores [35]. The specific surface areas (S_BET_) of the investigated materials were calculated using the Brunauer–Emmett–Teller (BET) theory. Table 1 shows that the pure CN sample had a characteristically low S_BET_ of only 17 m^2^/g. In contrast, all pure TiO_2_ components had higher S_BET_ values, with the highest value of 337 m^2^/g for the aTT sample, probably due to the nanotubular morphology observed in the TEM analysis (see below). All investigated composites exhibited higher S_BET_ values compared to the pure CN sample, regardless of the TiO_2_ morphology used. When the mortar composites were annealed, a slight decrease in the S_BET_ value can be observed, which is due to the partial melting of the CN component [36]. Since the pure aTT sample had a relatively high S_BET_ value, the composites retained this high value. Interestingly, the CNTP and CNTR series had similar S_BET_ values, although the pure TR sample had a higher S_BET_ value than the pure TP solid. The greater decrease in the S_BET_ of the CNTR series compared to the TR sample may indicate that a greater proportion of the CN component was present in the pores of TR than in the CNTP series, resulting in closer contact between the components. Using the Barrett–Joyner–Halenda (BJH) theory, we calculated the pore distribution (Figure S1b), which shows that most of the pores originated from the TiO_2_ component. It can be seen that the aTT-containing samples have three peaks compared to TR- (two peaks) and TP-containing samples (two peaks). The smaller mesopores allow the aTT and TR samples to have a larger surface area. The addition of the low porosity CN component decreased the intensity of all composite samples compared to the pure TiO_2_ samples. However, the BJH curves of the composites are still similar to the curves of pure TiO_2_. The most drastic decrease was observed for the CNTR/2 composite, as the pore volume decreased by almost 50% compared to the pure TR sample (Table 1), followed by the CNTP composite. This could indicate that a larger portion of the CN component penetrated into the pores and formed a tight bond with the TR component. Although the specific surface area was similar for the CNTP and CNTR series, the pore volume and pore diameter were slightly larger for the CNTR series, as the value was higher for the pure TR sample. This can be advantageous for the photocatalytic activity, as the molecules can reach the active sites more easily if the material is more porous.

#### 2.1.4. Other Surface Properties

The surface properties of the photocatalysts influences their photocatalytic reaction [37,38]. To obtain information about the acidic surface sites (AcSS) and the point of zero charge (pH_PZC_), we performed measurements of Pyr-TPD (Figure S2a,b) and the pH-related zeta potential (Figure S3a–d). It can be observed that the AcSS originates from the TiO_2_ component, with the highest AcSS concentration being 428 µmol/g_cat_ (TR, Table S2). The addition of the CN component decreased the AcSS concentration regardless of the synthesis method used, with the exception of the CNaTT/2 composite, where changes in aTT occur during calcination (Figure 2, XRD). Moreover, the AcSS density is similar for the annealed samples, so that the acidic surface properties probably do not play a decisive role in the photocatalytic process. Similar observations can be made in the determination of pH_PZC_. A detailed description of AcSS and pH_PZC_ can be found in the Supplementary Information.

#### 2.1.5. Investigation of Phase Composition, Morphology, and Crystal Structure

**Figure 3:**
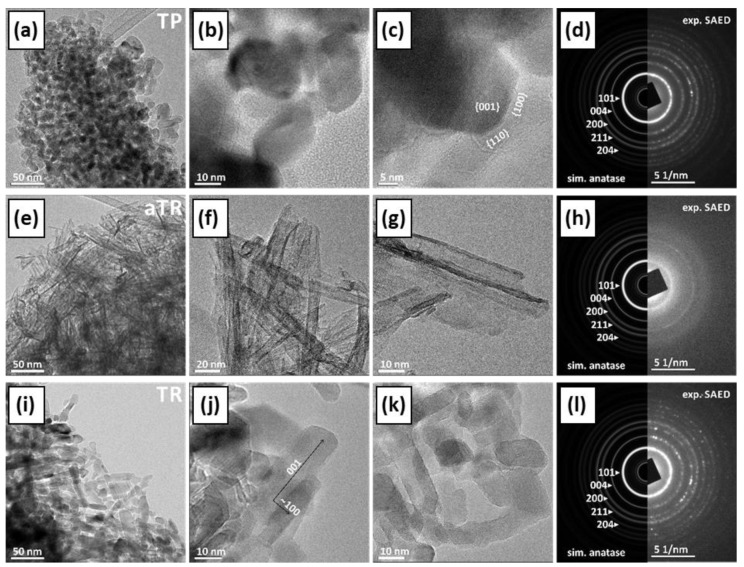
TEM micrographs of the investigated TiO_2_ morphologies ((**a**–**c**) TP, (**e**–**g**) aTT, and (**i**–**k**) TR) obtained at different magnifications, and corresponding experimental selected area electron diffraction (SAED) patterns compared with the simulated patterns for anatase ((**d**) TP, (**h**) aTT, and (**l**) TR)).

**Figure 4:**
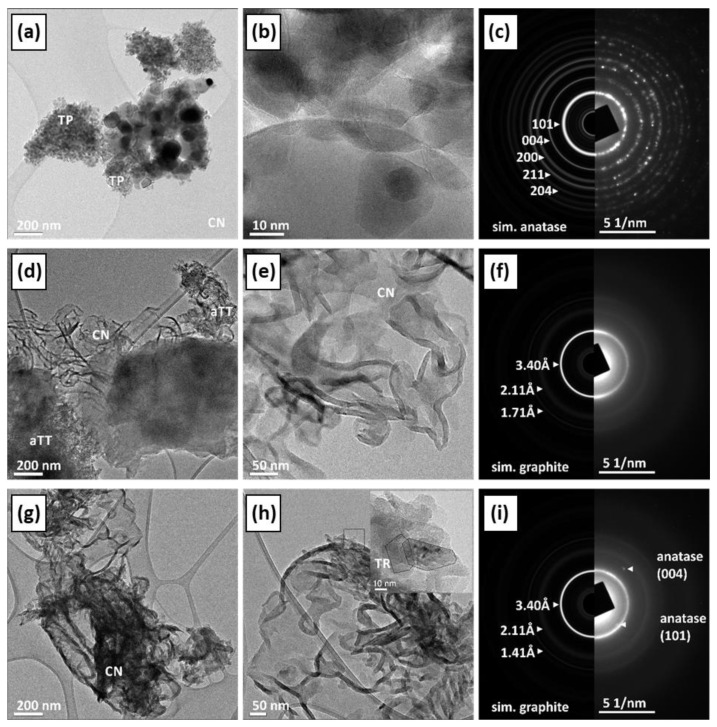
TEM micrographs obtained at different magnifications and results of experimental selected area electron diffraction (SAED) patterns compared with the simulation patterns for anatase and graphite for CNTP (**a**–**c**), CNaTT (**d**–**f**), and CNTR (**g**–**i**).

The phase composition, morphology, and crystal structure of the samples were analyzed using transmission electron microscopy (TEM) and scanning electron microscopy (SEM in Figure S4, see Supplementary Information). Figure S5a contains supporting information about the determination of the suggested indexation of the crystal facets. The commercial TiO_2_ sample (TP) consisted of elongated monocrystalline anatase particles with a ratio of approximately 3:1. Most particles are rounded, and some show typical {100} and {101} facets (Figure 3a–d). The aTT sample consists of poorly crystallized anatase nanotubes with a 2 nm thick wall and a diameter of ~10–20 nm, while the total length is >100 nm (Figure 3e–h). The TR sample consists of single crystalline anatase particles with a diameter of 10–20 nm and a length of up to 50 nm, which were probably formed by the collapse of the tubular aTT structure as a result of heat treatment at 500 °C. The particles are elongated in the c-axis direction (Figure 3i–l and Figure S5b). When examining the TiO_2_/g-C_3_N_4_ composites with the TEM microscope, we found that the CN phase is very sensitive to electron beams and tends to deteriorate under the examination conditions. The agglomerates are several µm in size, while the individual CN sheets have a diameter of up to 200 nm and are only a few nm thick. At the edges, the CN sheets tend to bend and form thicker and uneven edges. The SAED is similar to graphene/graphite, indicating a layered structure with a spacing of approximately 3.4 Å. The composites of TiO_2_ and CN show different degrees of interlocking depending on the synthesis method used. Both phases, TiO_2_ and CN, retained their typical morphology. In the calcined sample, we observed partial crystallite growth; a small amount of anatase particles (TP) showed a clear increase in size, while their morphology remained round, without characteristic crystal facets and without phase transformation (Figure 4a–c). In the samples mixed with mortar, the aTT nanotubes and CN films remain together with their morphology (Figure 4d–f). In addition to mixtures, it is possible to recognize an accumulation of individual aTT and CN phases, indicating incomplete mixing. In the TR sample, we found anatase particles up to 50 nm in size, which mainly accumulated at the thickened CN edges (Figure 4g–i). This could limit the photocatalytic activity as the total area of the interface is not optimal (TR evenly distributed over CN), which may limit the efficiency of the separation of charge carriers.

### 2.2. Optical and Electronic Properties of the Investigated Materials

#### 2.2.1. UV-Vis DR Measurements

**Figure 5:**
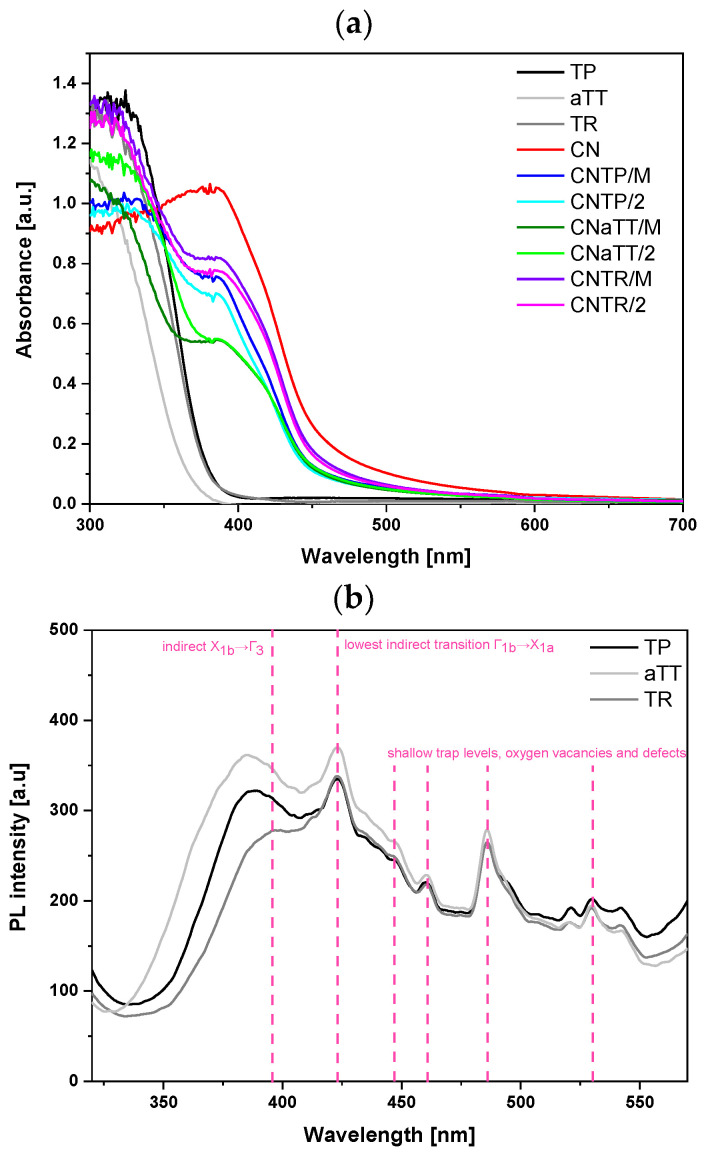

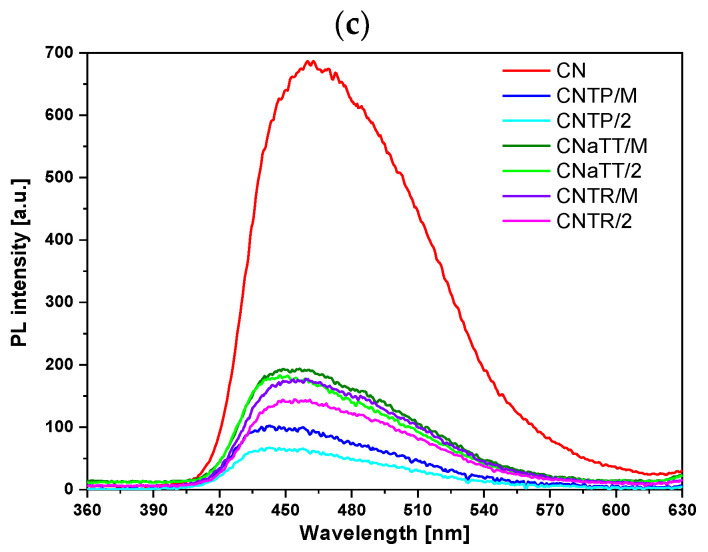
Results of (**a**) UV-Vis DR measurements, (**b**) solid-state photoluminescence (PL) spectra of bare TiO_2_ morphologies (excitation at 300 nm, scan rate 150 nm/min, emission slit 5.5 nm), and (**c**) bare CN and TiO_2_/CN composites (excitation at 320 nm, scan rate 200 nm/min, emission slit 2.6 nm). Pink dashed lines indicate characteristic TiO_2_ transitions.

We carried out UV-Vis DR measurements to evaluate the light absorption properties of the investigated materials. Figure 5a shows that all pure TiO_2_ components absorb light only in the UV light range. Due to the amorphous nature of the TiO_2_ in the aTT sample, the band edge was blue shifted compared to the TR and TP samples. In contrast, pure g-C_3_N_4_ absorbs UV light and light in the visible range almost up to 550 nm due to the π-conjugated system [4]. All investigated composites exhibited absorption in the visible range (up to nearly 550 nm) due to the presence of g-C_3_N_4_ and retained absorption features resembling both components. The range of visible-light absorption was largest for the CNTR series, followed by the CNTP series, which may indicate an improved photocatalytic activity of the CNTR series, as a larger portion of the visible-light spectrum can be utilized. The CNTR series also had a slightly changed absorption edge that might indicate the presence of a different kind of interaction (transition) between the CN and TR component. Using the Kubelka–Munk theory (Equation (1)), we calculated the optical band gaps of the investigated materials (Table S3). As expected, all pure TiO_2_ components exhibited characteristic band gaps for pure anatase titania, with the lowest value of 3.19 eV being for the TR sample. The lower value could be due to the presence of defects and/or the increased amount of Ti^3+^ affecting the light absorption properties [5,6,12,39,40,41]. The presence of defects and even some distortions [42] in the material can slightly shift the VB and CB to reduce the optical bandgap. However, the shift compared to the original TP (band gap of 3.23 eV) was small. This agrees with the XRD analysis, where we did not observe any major changes in the lattice for TP or TR samples as no obvious peak shifts were present. The presence of Ti^3+^ and O-vacancies (confirmed below) will have another effect for the light absorption properties, as they usually generate mid-level band gap defect states below the CB that lower the required energy barrier that photogenerated electrons need to overcome [5,6,12,39,40,41]. One could argue that the “effective” band gap is reduced in comparison to the “true” optical band gap. Therefore, we expect that pure TR and all CNTR composites will exhibit enhanced visible-light absorption and improved photocatalytic responses. These mid-level defect states are also probably the reason for the slight change in the shape of the absorption edge for all CNTR composites. The addition of CN (2.58 eV) decreased the band gap values for all composites. Since the band gap value was highest for the aTT sample, the composites also exhibited a higher value compared to other composites (Table S3). When all mortar composites were annealed for 2 h, a slight increase in the band gap value was observed as the g-C_3_N_4_ decomposed slightly (Table S1), except for the CNaTT/2 composite, where the crystallinity of the aTT component was improved during annealing. However, a slightly higher band gap can also have positive effects on the catalytic activity, as it can hinder the recombination of charge carriers.

#### 2.2.2. Solid-State PL Measurements

To assess the radiative charge recombination tendency, we measured the solid-state photoluminescence (PL) spectra of the samples. Figure 5b shows the PL spectra of the pure TiO_2_ components, with an experimental setup different from the g-C_3_N_4_ samples. The PL signals were similar across TiO_2_ morphologies. The first transition (~3.17 eV) corresponds to the band edge of anatase TiO_2_ (indirect transition X_1b_→Γ_3_ [43,44]). The TR sample, having the lowest band gap, shows the most red-shifted edge transition at 395 nm (3.14 eV). As the band gap increases, this transition shifts blue to 387 nm (TP) and 384 nm (aTT). TR also exhibits the lowest PL intensity, indicating favorable carrier dynamics and reduced charge recombination, potentially due to its larger particle size compared to TP [45]. The peak at 423 nm corresponds to the lowest indirect transition Γ_1b_→X_1a_ (2.93 eV). The aTT sample shows the highest PL signal due to its low crystallinity, which promotes charge recombination, though no peak shift is observed. Similarly, the peaks at 446, 460, 485, and 529 nm, caused by shallow trap levels, oxygen vacancies, and defects, did not shift [8,43,44,45,46]. These oxygen vacancies likely formed during TiO_2_ synthesis. The hydrothermal synthesis and subsequent calcination of aTT to TR increase defects (oxygen vacancies), potentially enhancing photocatalytic activity [47]. This is reflected in the reduced PL signal at 529 nm (2.34 eV) compared to TP. Moderate defects act as traps, preventing charge recombination, while excessive defects accelerate it.

We also measured the solid-state PL spectra of the composites with corresponding settings for g-C_3_N_4_, the component active in visible-light absorption. Figure 5c shows that all photocatalysts exhibit a broad peak at ~460 nm (2.64 eV), which can be divided into three peaks using a Gaussian model fit: σ*-LP (440 nm), π*-LP (460 nm), and π*-π (490 nm) [4,25,30,48], as seen in Figure S6a–d. Comparing pure CN and composites reveals two changes (Table S4): (i) a slight blue shift due to heterojunction formation and (ii) a drastic decrease in PL intensity (area). The PL intensity reduction is mainly attributed to fewer π* → π and π* → LP transitions in g-C_3_N_4_ [25], as photogenerated electrons move from the conduction band (CB) of g-C_3_N_4_ to the CB of TiO_2_, regardless of morphology. Composites with aTT have lower crystallinity, reducing charge carrier mobility, which accounts for the highest PL intensity among the composites. Increased TiO_2_ crystallinity improves the charge carrier mobility, leading to a lower PL signal. Annealing mortar composites further enhance the charge carrier transport by improving the interface between components. The CNTP/2 composite had the lowest PL signal, likely indicating the longest charge carrier lifetime, as all transition areas decreased significantly alongside a peak blue shift compared to CN. Meanwhile, CNTR/2 showed a larger area for all three peaks and the greatest red shift among composites. However, a blue shift in CNTR/2 compared to CN indicates heterojunction formation. Differences in peak positions between CNTP/2 and CNTR/2 may suggest distinct interactions between CN and TP or TR components. This was also evident in UV-Vis DR spectra (Figure 5a), where CNTR series absorbed the most visible-light. The SEM images (Figure S4b) show that TiO_2_ is more exposed on g-C_3_N_4_ in the CNTP composite, possibly reducing the PL signal since PL spectra measurement settings were optimized for g-C_3_N_4_. The exposed TiO_2_ could act as a shield, distorting results. The UV-Vis DR, EPR, and XPS analyses revealed surface Ti^3+^ in TR and CNTR/2 samples, which can generate charge carriers under visible-light. In CNTR/M and CNTR/2 composites, this enables a Z-scheme mechanism, where *h^+^* from CN and e- from TR recombine, producing a PL signal. This explains the higher PL signal of CNTR/2 compared to CNTP/2, which has little or no Ti^3+^ content.

#### 2.2.3. Electrochemical Investigation

Normally, the lowest PL signal indicates the best photocatalytic performance, as the chances of utilizing charge carriers increase. However, this assumption might be wrong, as charge carriers can also recombine non-radiatively. Therefore, we performed electrochemical impedance spectroscopy (EIS) to evaluate the charge transfer resistance (R_CT_) and to obtain information about the dynamics of charge transfer in the investigated materials. The obtained Nyquist diagrams in Figure S7a–d were fitted with the electrochemical equivalent circuit shown in Figure S7e. From the results in Table S3, all pure components exhibit high R_CT_ values, which limits their photocatalytic activity. Of all the pure TiO_2_ components, the TR sample exhibited the lowest R_CT_ value (0.90 MΩ), which can be attributed to the improved structural and optoelectronic properties described in the previous sections (and below in the EPR section). In comparison, all composites exhibited lower values than their pure counterparts, as the formation of a heterojunction improves the charge transfer dynamics. Due to the poor crystalline nature of the aTT sample, all composites with aTT retain a high R_CT_ value. Since the properties of the CNaTT/2 composite are similar to those of the CNTR/M material, it is no wonder that they have similar R_CT_ values. The lowest R_CT_ value of all photocatalysts, i.e., the best charge carrier transfer, is exhibited by the CNTR/2 photocatalyst (0.40 MΩ), followed by the CNTP/2 composite (0.48 MΩ). In general, the entire CNTR series shows better charge carrier dynamics than the CNTP series. This is due to the unique properties of the TR sample, such as the improved crystallinity and the higher Ti^3+^ content at the surface (see EPR and XPS section below). The latter could enable the formation of an intimate heterojunction between g-C_3_N_4_ and TiO_2_ and influence the nature of the charge carrier mechanism in the photocatalytic reactions. Overall, the EIS results agree quite well with the PL measurements. However, since they account for all recombination processes, they confirm that charge separation is most enhanced in the CNTR/2 photocatalyst. Consequently, we can assume that the CNTR/2 photocatalyst could be the most active among all composite materials.

#### 2.2.4. Solid-State EPR Investigation

To investigate the properties of the synthesized materials, we performed direct CW-EPR measurements at room temperature (RT) and liquid nitrogen temperature (LN2). All samples were measured in an air atmosphere. The solid-state EPR spectra recorded at RT (Figure S8) show that different TiO_2_ morphologies yield distinct EPR spectra, with signals for O-vacancies, Ti^3+^, N-species, S-species, etc. [49,50,51,52,53]. Due to the amorphous nature or low crystallinity of the aTT sample, it likely lacks paramagnetic centers, resulting in an EPR-silent signal in both the dark and under visible-light illumination (Figure S8). The exception is the signal at a g value of 2.004, which could be attributed to CB electrons or O-species formed by hole trapping in the subsurface [52,53,54,55,56]. The g-values above 2.0 typically indicate hole trapping (O-vacancies interacting with O-species), while values below 2.0 correspond to electron trapping (at Ti^3+^ sites in the bulk or surface). The absence of Ti^3+^ and O-vacancy signals in the aTT sample can be due to the limitations of RT EPR measurements, which affect the resolution [55,57]. Furthermore, due to the EPR spectrometer’s limitations and sample heterogeneity, some signals may merge or be lost, increasing uncertainty. In contrast, the TP and TR samples show an EPR response in the dark (Figure S8), attributed to O-vacancies (hole trapping) and Ti^3+^ (electron trapping) [49,50]. Both samples exhibit the g = 2.004 signal observed in the aTT sample. In the TP sample, we observe sharp signals for Ti^3+^ and O-vacancies, with a peak at g = 2.007 and a small shoulder, suggesting similar O-vacancies without complex environments. The signal at g = 2.023 may also belong to this group, alongside the g = 2.007 and g = 2.004 signals for O-species [12,49,52,54,58,59,60]. The signal at g = 2.023 could originate from paramagnetic sulfur species, as the CHNS analysis showed a low sulfur content (<0.1 wt.%), likely due to the synthesis procedure [51]. We also observe a signal at g = 1.997 for the TP sample, attributed to Ti^3+^ in the bulk/lattice, along with other Ti^3+^ signals at g = 1.981, g = 1.969, g = 1.956, and g = 1.943 [49,59], although their resolution is low. The signals at g = 1.997 and around 1.960 likely represent bulk Ti^3+^ [12,52,56,57,58,61,62], as suggested by the shape of the g = 1.997 signal [48]. Other signals overlapping between 340 and 355 mT may correspond to surface Ti^3+^ in different environments, though their concentration is minimal, as no surface Ti^3+^ was detected using XPS (see below). Since XPS is a surface-sensitive technique with a limit of detection (LOD) of about 0.1 at.%, we conclude that most Ti^3+^ in the TP sample is in the bulk/lattice. Furthermore, the UV-Vis DR spectra and the color of the TiO_2_ samples (remaining white) suggest that the Ti^3+^ content is minimal. Thus, distinguishing specific features in the 340–355 mT range using X-band CW-EPR at RT is challenging. Under visible-light illumination, the TP sample shows no electron capture at g < 2.0, likely due to the low Ti^3+^ content in the bulk/lattice, which cannot generate charge carriers. However, in the g > 2.0 region, signals corresponding to hole trapping are observed. The signal at g = 2.004 decreases, while those at g = 2.023 and g = 2.007 increase, with g = 2.007 showing the most significant increase, likely due to hole trapping at O-vacancies (defects) [10,47]. In contrast, the TR sample exhibits a different EPR spectrum, with signals for O-species at g = 2.004 and g = 2.007, along with a more pronounced O_2_^−^ signal at g = 2.095. The g = 2.023 signal is absent in the TR sample, and, since neither the TR nor aTT samples show the g = 2.023 signal, and no sulfur impurities were detected in their XPS spectra, we can tentatively attribute the g = 2.023 signal to sulfur impurities removed during hydrothermal synthesis. It is unusual for the TP sample to show a signal for O_2_^−^ species, as most O-vacancies and defects are present in the bulk at low levels, making it difficult for oxygen to interact with holes. However, the TR sample likely has a higher number of O-vacancies on the surface and subsurface, which could generate this signal. Additionally, the peak at g = 2.007 becomes broader and more intense, suggesting more surface and subsurface O-vacancies that create different environments [54,57,58,60]. This is supported by solid-state PL measurements of bare TiO_2_, indicating a higher O-vacancy content. When comparing the TP and TR samples, we observe changes at g < 2.0, specifically the disappearance of the signal at g = 1.997, corresponding to bulk Ti^3+^. This suggests that Ti^3+^ is more present at the surface than in the bulk/lattice, likely due to hydrothermal treatment and crystallization at 500 °C. Some broadening of the signal could merge with O-vacancy signals, indicating more Ti^3+^ at the surface [50,52]. The XPS measurements also confirmed the presence of surface Ti^3+^. Moreover, we see an increase in the broad signal between 340 and 350 mT, confirming a higher proportion of surface Ti^3+^ in the TR sample, which is favorable for visible-light absorption. The Ti^3+^ forms defect states in the band gap below the CB, allowing for visible-light utilization. The more defect states present on the surface, the more visible light can be used. These observations are also reflected in the red-shifted UV-Vis DR absorption edges of TR-containing samples. This means more charge carriers can interact with oxygen species in water to form ROS. Under illumination, the TR sample showed no major changes at g < 2.0, likely due to thermal effects at room temperature and fast charge carrier recombination, but we observed an increase in EPR signals at g > 2.0. In contrast to the TP sample, the TR sample showed an increase in all signals corresponding to oxygen species, indicating successful hole trapping at O-vacancies. This is why the TR and CNTR samples exhibit a lower PL and the lowest R_CT_ values compared to other materials.

**Figure 6:**
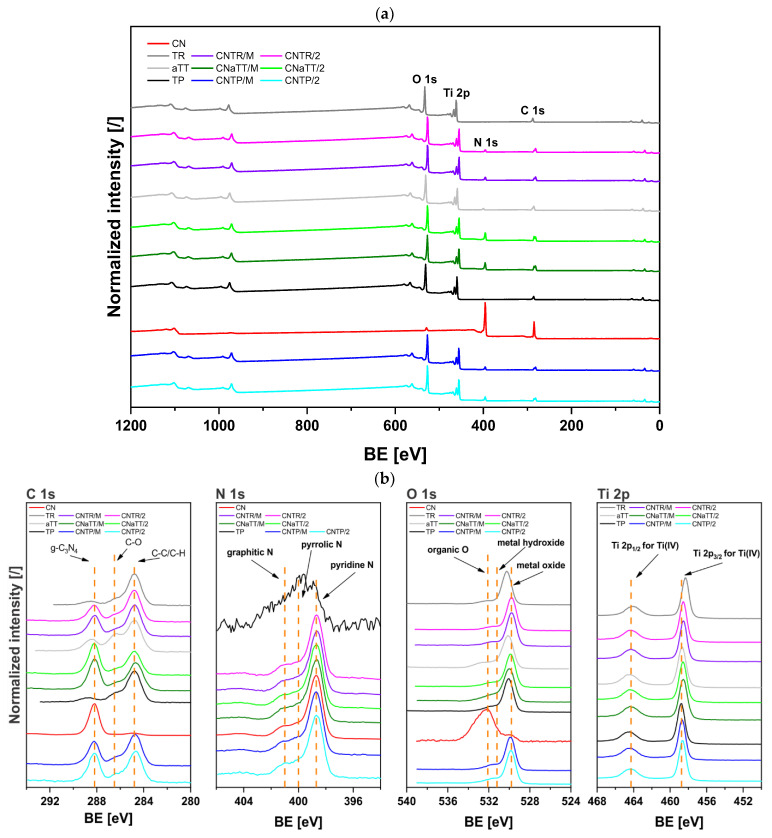
(**a**) Survey and (**b**) high-resolution XPS spectra for the investigated catalysts samples.

To fully evaluate the properties of all TiO_2_ components, we performed CW-EPR measurements at liquid nitrogen (LN2) temperature. As seen in Figure S9, the aTT sample still shows no characteristic signals for Ti^3+^ or O-vacancies, only the O- signal at g = 2.004. Similar to the RT measurements, the TR sample exhibits signals for O-vacancy sites (g > 2.000), O- (g = 2.004), and Ti^3+^ in the featureless region around g = 1.934. The small features in the RT spectra of TR (Figure S8) are likely artefacts or thermal interactions related to electron capture (Ti^3+^ at g < 2.000). The spectra suggest that Ti^3+^ and most O-vacancies in TR are located on the surface, with O-vacancies also present in subsurface levels. No signals for nitrogen, carbon, or sulfur species were observed in the TR or aTT samples, confirming that the hydrothermal treatment removed impurities. This is also supported by XPS data, which show no S or N signals, and only a small amount of adventitious carbon species on the aTT and TR surfaces. The aTT sample has more adventitious carbon due to its large specific surface area. Surprisingly, the LN2 EPR spectra of the TP sample show the greatest difference compared to its RT spectra, and to the spectra of aTT and TR samples. The signals are intense, sharp, and well-defined. These signals likely correspond to nitrogen species on the surface of the TP sample, as observed in the XPS survey spectra (Figure 6a). This allows for a clearer assignment of the different EPR signals for the TP sample compared to the RT measurements, where the error margin was larger. From Figure S9, we observe two different nitrogen species, although the resolution is limited due to the EPR spectrometer and sample heterogeneity. Several signals were identified, including nitrogen species (NO) adsorbed on the surface, marked with blue, green, and magenta in Figure S9. Notably, signals at g = 2.002 and g = 1.979 were absent in the RT EPR spectra, consistent with findings in the literature [63,64]. Additionally, signals corresponding to a single nitrogen atom trapped in the bulk of TiO_2_ (Nb) were detected, labeled olive, red, and dark orange. The signals at g = 2.006 and g = 2.022 could also be attributed to O- and S-species [51,53], though distinguishing between them is challenging due to signal overlap and sample heterogeneity. However, some paramagnetic nitrogen species may still be present in the bulk, in agreement with previous studies [62,63,64,65]. Our alkaline hydrothermal treatment removed these nitrogen and sulfur impurities while “rebuilding” the TiO_2_ nanoparticles. Thus, TR and aTT materials show no N-species signals. We also detected two signals likely corresponding to Ti^3+^ in the bulk/lattice (g < 2.000) [50]. The LN2 EPR results differed slightly from RT data but confirm that (a) the TP sample was purified from nitrogen and sulfur impurities, (b) the surface and subsurface O-vacancies in TR were increased, and (c) a small amount of surface Ti^3+^ was introduced into the TR sample, enhancing the photocatalytic activity of the CNTR series.

We also performed CW-EPR measurements at RT for pure g-C_3_N_4_ (CN) and CN-containing samples. For pure CN (Figure S10a), we observe a characteristic single Lorentzian line with a g value of ~2.007, typical of g-C_3_N4 due to unpaired electrons trapped at sp2 C atoms in the heptazine moiety [66]. Upon visible-light illumination, the signal intensity increases as photogenerated electrons act as paramagnetic centers. The signal broadens at higher magnetic fields, likely due to defect sites and different environments in pure CN. When comparing the EPR spectra of the composites (Figure S10b,c), the CNTR series (both mortared and annealed for 2 h) shows the highest EPR signal/area compared to the other composites, with CNTP/2 being similar to CNTR/2. These results align with the PL and EIS analyses (Figure 5c and Figure S7), where the CNTR and CNTP series exhibited good carrier dynamics. In the EPR spectra of all composites, we observe an intense signal at g ≈ 2.007, corresponding to the CN component. The different shapes of CNTR/M and CNTR/2 suggest the presence of O-vacancies and possibly Ti^3+^ from the TR component, which has a higher quantity of surface O-vacancies and Ti^3+^ than the TP component. Since the defects visible in the EPR spectra act as traps, charge carrier recombination is hindered, resulting in a decrease in the PL signal (Figure 5c) and a reduced R_CT_ value (Table S3). However, for effective photocatalysis, these traps must be superficial. If deep, electrons are captured but cannot generate reactive oxygen species (ROS). To validate these EPR observations, we performed XPS analysis and photocatalytic experiments under visible light (discussed below).

### 2.3. XPS Analysis of Solid Nanomaterials

Figure 6 shows the survey and high-resolution XPS spectra. The survey spectra in Figure 6a show signals for C 1s, N 1s, O 1s, and Ti 2p. The Ti 2p signal is absent in the CN sample because it contains only pure g-C_3_N_4_. The N 1s signal is missing in the aTT and TR samples, likely due to hydrothermal synthesis and calcination in air at 500 °C, which removed surface-adsorbed N-containing species. The TP sample also shows peaks for S 2p and S 2s, indicating sulfur residues from the manufacturer’s synthesis process, which were removed using hydrothermal treatment in the aTT and TR samples. The Na 1s peak at about 1072 eV is absent, confirming that no sodium was present in our materials. The Na KLL Auger peaks at about 500 eV are also missing. XRD and SAED analysis further supports this, showing no detectable Na species, confirming that no Na titanate was retained in the dried aTT or TR samples despite hydrothermal treatment with NaOH.

High-resolution C 1s spectra are shown in Figure 6b. The spectra consist of three contributions: a C-C/C-H peak at 284.8 eV, a high-intensity shoulder on the high binding energy side representing C-O, and the peak for C in g-C_3_N_4_ at 288.2 eV (example in Figure S11a) [32]. The C-C/C-H and C-O peaks are from adventitious carbon. The C in g-C_3_N_4_ peak is absent in the TP and TR samples because they lack g-C_3_N_4_. The CN sample shows only the C in the g-C_3_N_4_ peak. In the aTT sample, the intensity of C-O species is higher compared to C-C/C-H, likely due to its higher specific surface area, providing more adsorption sites. Overall, the C 1s spectra of all composites show similar shapes and positions, indicating a comparable C environment regardless of the TiO_2_ support or synthesis method.

The N 1s spectra are shown in Figure 6b and can be fitted with three components, as shown in Figure S10b. The signal at 398.7 eV is assigned to pyridine-N, followed by pyrrole-N at 400.0 eV, and the peak with the highest binding energy is assigned to graphitic N [32] (example in Figure S11b). A lower intensity peak is observed in the TP sample, likely due to the manufacturer’s synthesis procedure or surface-adsorbed nitrogen species. Similar to the C 1s spectra, all composites show comparable N 1s spectral shapes and positions, indicating a similar N environment.

The high-resolution O 1s XPS spectra (Figure 6b) show three features: Ti oxides at ca. 529.8 eV, a weak shoulder for metal hydroxides at ca. 531.2 eV, and a peak with the highest binding energy (intense in the CN sample), likely from organic compounds and/or water molecules on the surface [32]. An example of a deconvoluted O 1s spectrum is shown in Figure S11c. In the TP sample, the signal appears at the lowest binding energy, shifting slightly to a more positive value for the TR sample. This shift suggests oxygen vacancy formation, but it may also indicate different types of -OH groups on the surface, as noted by Posada-Borbón et al. [67]. The positive shift should be interpreted with caution. Solid-state EPR and PL measurements, however, indicate a higher content of oxygen vacancies in the TR sample. There are no major differences in the chemical environment between the photocatalysts, as seen in the C 1s and N 1s spectra.

High-resolution Ti 2p XPS spectra are shown in Figure 6b. The Ti 2p_3/2_ peak for Ti^4+^ [32] appears at a similar binding energy for all samples, except for the TR sample, where it shifts to a more negative binding energy, indicating more Ti^3+^ on the surface due to oxygen vacancy formation [68,69,70]. This shift is supported by solid-state EPR measurements. For the TR sample, the increased oxygen vacancy content (EPR and PL) also correlates with higher Ti^3+^ levels observed in XPS. No Ti^3+^ is seen in the TP sample, as Ti^3+^ is deeper in the material, as confirmed using EPR and XPS [6]. Furthermore, the lack of a shift of the Ti 2p_3/2_ peak to more positive binding values, compared to TiO_2_, confirms the absence of NaTiO_3_. Comparing the CNTP/2 composite with the pure TP sample, there was a slight shift to a more negative binding energy. However, for the CNTR/2 composite, a shift towards a more positive binding energy was observed, suggesting a different charge carrier mechanism, possibly a Z-scheme similar to Jo et al. [30]. Still, the binding energy of CNTR/2 was slightly lower than CNTP/2, likely indicating a higher surface Ti^3+^ content in the CNTR/2 sample.

### 2.4. Photocatalytic Activity Under Visible-Light Illumination

#### 2.4.1. Photocatalytic Bisphenol A Degradation

**Figure 7:**
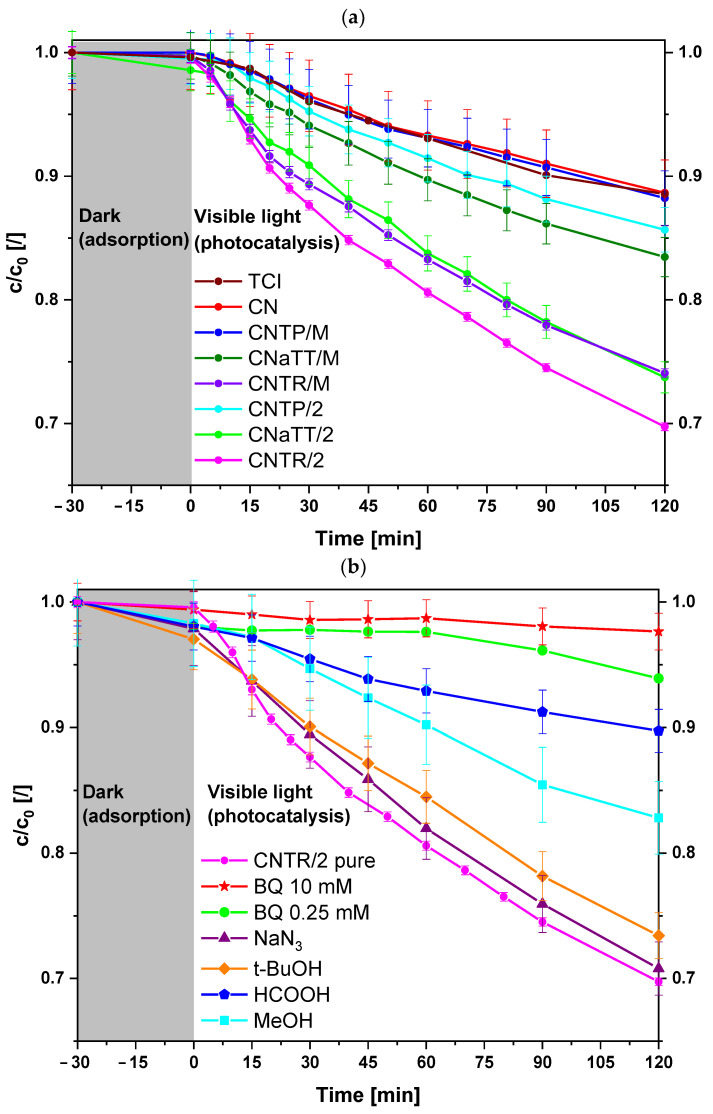
(**a**) Photooxidation of water-dissolved bisphenol A (BPA) in the presence of the investigated photocatalysts under illumination with visible light and (**b**) in situ quenching experiments for CNTR/2 sample. The gray area represents the dark period in which the adsorption–desorption equilibrium is formed. The average ± standard deviation of triplicate tests is shown.

In order to evaluate the investigated photocatalysts for use in possible environmental remediation processes, the photooxidation of dissolved BPA in water was carried out under visible light (Figure 7a and Figure S12a). Due to the high band gap value of pure TiO_2_, the aTT, TP, and TR materials exhibited only low BPA degradation (Figure S12a), which was mainly due to the presence of some defects. This is especially true for the TR sample, where the higher content of Ti^3+^ allows some response to visible light, as the surface Ti^3+^ and O-vacancies induce mid-level defect states [10]. Due to the low specific surface area and the high tendency to recombine charge carriers, the pure CN also shows a low BPA degradation rate under visible light (Figure S12a). On the other hand, all CN-containing photocatalysts show an improved photocatalytic response when illuminated with visible-light (Figure 7a). Since the gray area in Figure 7 represents the dark period in which only adsorption/desorption processes take place, we can conclude that no adsorption takes place, as the maximum value does not even reach 3%. Therefore, the main mechanism responsible for the removal of BPA is photocatalysis. Interestingly, the activity of the CNTP/M composite is still low and improves when additional calcination is performed to form a better heterojunction (as seen in PL and EIS analysis). Nevertheless, it is lower than that of the CNaTT and CNTR series. Due to the increased S_BET_, the CNaTT/M and CNaTT/2 composites show a better response than the CNTP series. Since annealing the aTT component changes the crystallinity (CNaTT/2), the photocatalytic response is similar to that of the CNTR/M material. The CNTR/2 sample shows the highest photocatalytic response, as the additional calcination improves the charge transfer process compared to the CNTR/M composite (PL and EIS), even though it had a lower S_BET_ than the CNaTT series. The CNTR/2 sample also had a significantly higher photocatalytic activity compared to commercial TiO_2_ (TP sample, Figure S12a) and g-C_3_N_4_ (TCI sample, Figure 7a). In addition, the CNTR/M and CNTR/2 composites showed high TOC removal with only low carbon accumulation (Table 2 and Table S1). We also found that the catalyst concentration used (125 mg/L) was likely optimal given the cost of the catalyst, as doubling the concentration (c_cat_ = 250 mg/L) only increased the activity by ~20% of BPA degradation (Figure S12b). The CNTR/2 composite maintained its high activity after three cycles (BPA degradation from 30.3 to 28.8%), as its structure did not change significantly before and after the reaction (Figure S12c,d), indicating increased stability.

In addition, the photodegradation efficiency of BPA by CNTR/2 was evaluated in comparison with other similar photocatalysts reported in the literature [36,71,72], as summarized in Table 3. The results show that the synthesized CNTR/2 composite exhibits comparable or even better performance in the degradation of BPA under visible light. This underlines the exceptional potential of CNTR/2 as an effective and promising photocatalyst for the degradation of BPA in an aqueous environment.

#### 2.4.2. In Situ Quenching Experiments

To find an explanation for the increased photocatalytic activity, we performed an in situ quenching reaction for the CNTR/2 composite (Figure 7b). We used the following quenchers: p-benzoquinone (BQ, $O_{2}^{-}\cdot$), sodium azide (NaN_3_, ^1^O_2_), tert-butanol (t-BuOH, OH·), and formic acid (HCOOH, *h^+^*). Figure 6b shows that quenching with NaN_3_ or t-BuOH has no effect on the photooxidation of BPA, which means that ^1^O_2_ and OH· are not the most important ROS. However, when using another OH· quenching agent, namely methanol (MeOH), we can observe a stronger decrease in activity. The reason for this could be that when t-BuOH is used, a certain amount of ROO· can be formed, which is used to degrade organic pollutants [73]. This could explain why we still observe high BPA degradation even when we intercepted OH·. However, for both OH· quenchers, the loss of activity is low, implying that OH· is not the dominant active species. The addition of BQ completely inhibits the degradation of BPA, which proves that the major ROS is $O_{2}^{-}\cdot$. In addition, the addition of HCOOH reduces the degradation of BPA to some extent, implying that the holes generated by the light also play an important role. Thus, the reason for the improved activity of the CNTR/2 composite must be related to the differences between the two TiO_2_ samples (TP and TR) that influences the formation of different ROS.

### 2.5. Discussion of Improved Photocatalytic Activity

**Figure 8:**
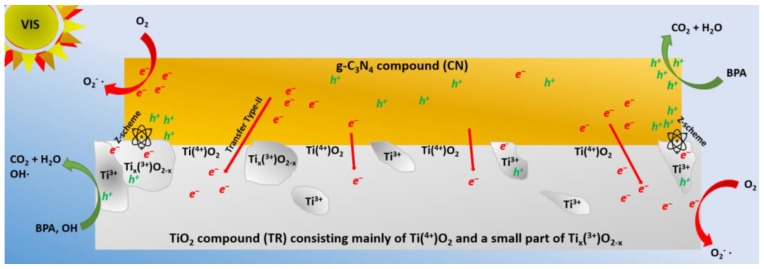
The proposed dual mixed type-II/Z-scheme charge carrier mechanism for the CNTR/2 photocatalyst under visible light. The gray area shows TiO_2_ with Ti(^4+^)O_2_, the main part of the TR particle, which does not produce charge carriers under visible light and follows a type-II transfer mechanism. The white areas represent small Ti_x_(^3+^)O_2−x_ regions that are excitable to visible light due to mid-level defects (Ti^3+^ and O-vacancies). The charge carrier formation in the TR component enables a direct Z-scheme mechanism.

The superior activity of the CNTR/2 composite can be attributed to several factors. First, the S_BET_ of the TP and TR series was similar, so it is not a decisive factor. The main difference lies in the better crystallinity of the TR series, which improves electron migration, and the elongated nanoparticle shape. In contrast, the aTT component, with poor crystallinity, reduces charge carrier mobility, despite its high S_BET_ and tubular morphology, which typically enhances photocatalytic activity. As a result, the CNaTT series shows lower activity than the CNTR series. Specifically, the CNTR/2 photocatalyst demonstrates the best charge carrier separation (lowest R_CT_ value of 0.40 MΩ). Second, the CNTR series has the largest pore diameter, allowing better access to active sites for BPA molecules. A larger pore size also reduces photon scattering and reflection, enabling greater light absorption [74]. Third, the decrease in S_BET_ between the CNTR/2 and TR samples is more pronounced than between CNTP/2 and TP, suggesting that the CN component interacts more strongly with the TR component, forming a closer interface. Fourth, the opto-electronic properties of the TR series differ from the TP series. Pure TR and its composites show a red shift in UV-Vis DR and PL measurements, indicating better visible-light absorption. This is due to the increased presence of surface Ti^3+^ and O-vacancies, observed in EPR and supported by the XPS analysis. Ti^3+^ enables visible-light utilization, while O-vacancies improve the charge separation in TR compared to other TiO_2_ materials (PL, Figure 5b) and CNTR composites (EIS, Table S3). Ti^3+^ and O-vacancies form defect states below TiO_2_ conduction band, reducing the energy required to form charge carriers and enhancing carrier separation by trapping them. The presence of Ti^3+^ in the TR sample and its ability to absorb visible light likely influences the type of heterojunction and migration mechanism. Typically, pristine TiO_2_ and g-C_3_N_4_ systems behave as type-II heterojunctions under visible light [75], since anatase TiO_2_ cannot absorb visible light. This was observed in the CNTP/2 composite, which behaves as a type-II heterojunction. However, when Ti^3+^ is present on the surface of TiO_2_ (as seen in EPR and XPS analyses), it can absorb visible light and generate charge carriers in the TiO_2_ component. In this case, the g-C_3_N_4_/TiO_2_ composite may behave as a direct Z-scheme photocatalyst, where the less reductive electrons from one component (TR) interact with the less oxidative holes in the other (CN), leaving the stronger counterparts intact. This is suggested by the XRD, PL, EIS, and XPS results [30]. Additionally, the CNTR series showed a higher PL signal intensity compared to the CNTP series. This could be due to shielding by TiO_2_ in CNTP, or the recombination of weaker charge carriers in the direct Z-scheme, which generates the PL signal. The spatial separation of stronger charge carriers is also reflected in the lowest R_CT_ value of the CNTR/2 composite. However, despite the ability of TiO_2_ to absorb some visible light, the absorption properties remain limited by the small amount of Ti^3+^ in the sample, as evidenced by the TR sample’s white color (instead of turning gray, black, or blue, as seen with higher Ti^3+^ amounts) [10]. It is likely that a dual charge carrier mechanism is at play [76,77,78,79,80,81,82,83,84,85]. Therefore, we can assume that both type-II and Z-scheme mechanisms are present in the CNTR/2 sample (Figure 8). The partial Z-scheme mechanism enhances photocatalytic activity via reduction electrons in CN and oxidative holes in the TR component (Ti^3+^ moiety), while the type-II process dominates in the majority of samples.

#### Proposed Charge Transfer Mechanism

In the proposed dual mixed charge transfer, the entire CN component produces charge carriers together with small areas of the TiO_2_ surface containing Ti^3+^ (Ti_x_(^3+^)O_2−x_). Most of the TiO_2_ particles (with Ti^4+^) do not produce charge carriers. According to the Z-scheme mechanism, the less reductive electrons in Ti_x_(^3+^)O_2−x_ parts recombine with the less oxidative holes in the CN component. This probably leads to the radiative relaxations observed in the PL measurements (Figure 5c). In the Ti_x_(^3+^)O_2−x_ part, the stronger holes remain, which can either generate OH· or oxidize BPA directly. The possible generation of OH· was observed in the coumarin scavenging experiments (see below) and the in situ BPA quenching measurements. On the other hand, the more reductive electrons remain in the CN component and can generate $O_{2}^{-}\cdot$ from O_2_ dissolved in water. However, since the fraction of Ti_x_(^3+^)O_2−x_ is low (only a few spots in the TiO_2_ particles), most of the electrons in the CN component jump to the CB of Ti(^4+^)O_2_, as this is energetically favorable according to the type-II mechanism. As a result, they have a lower reduction potential, which limits the overall activity of the CNTR/2 sample, as most of the material behaves like a type-II. Furthermore, as can be seen from the TEM analysis, the TR particles are mostly agglomerated at the edges of the CN sheets, which further limits the charge carrier transfer dynamics. Nevertheless, the dual mixed scheme CNTR/2 composite shows a significant improvement in photocatalytic activity compared to the pure type-II mechanism of the CNTP/2 composite. These differences, coupled with the moderate S_BET_, enabled the CNTR/2 composite to show enhanced photocatalytic activity in the visible light-assisted photooxidation of BPA.

### 2.6. Reactive Oxygen Species Scavenging Under Visible-Light Illumination

#### 2.6.1. Determination of Hydroxyl Radical Generation Tendency

To evaluate the proposed mechanism of electron migration from Figure 8 and the in situ quenching experiments (Figure 7b), we first performed coumarin scavenging experiments under visible light to determine the generation of OH· (Figure S13a). Since the valence band (VB) of g-C_3_N_4_ is too positive to form OH· directly, they are generated via the superoxide radical anion ${\;O}_{2}^{-}\cdot$, which forms H_2_O_2_ that decomposes to OH·. However, as assumed in Figure 8, a low concentration of OH· can be formed directly from the *h^+^* in the Ti_x_(^3+^)O_2−x_ part of TiO_2_ particle. From Figure S13a, it can be seen that pure CN produces the least OH·, as it has a low S_BET_ and high carrier recombination (highest PL signal intensity and R_CT_ value). The addition of TiO_2_ enhanced the photocatalytic reaction due to the injection of *e*^−^ from the CB of CN into the CB of TiO_2_, regardless of the morphology of TiO_2_. Alternatively, as in the case of the CNTR series, a partial Z-scheme mechanism can proceed. The enhancement of the injection depends on the close interface between the components [25]. As mentioned above, the mortar series exhibits a non-optimal heterojunction, resulting in a lower photocatalytic activity compared to the annealed version. In addition, the CNaTT/M composite produces the lowest amount of OH·, which can be attributed to the poor crystalline nature of the aTT component that limits the mobility of the charge carriers. Interestingly, both the CNTP/2 and CNTR/2 samples showed similar OH· formation rates, with a slight increase in the latter. This could be due to the presence of *h^+^* in the Ti_x_(^3+^)O_2−x_ portion of the TiO_2_ particle in the CNTR/2 sample. However, since the Ti_x_(^3+^)O_2−x_ part is the smaller part of the overall TiO_2_ particle, most of the charge transfer is subject to the type-II mechanism, limiting the formation of ROS, in this case OH·.

#### 2.6.2. EPR Spin Trapping Experiments

Since *e*^−^ plays an important role in the use of g-C_3_N_4_ materials and to confirm the increased reduction potential of *e*^−^ in the CB of CN, we performed in situ EPR spin trapping with DMPO/DMSO/catalyst-aerated suspensions to follow the generation of ${\;O}_{2}^{-}\cdot$. Figure S13b shows that pure DMSO or DMPO/DMSO does not generate a signal in the dark or when illuminated with visible light. This also applies to CNTP/2 and CNTR/2 suspensions in the dark. However, upon illumination with visible light, we observed the presence of a 12-line signal. The spectra are dominated with the characteristic signals of the DMPO-${\;O}_{2}^{-}\cdot$ adduct for both photocatalysts with signal splitting due to interactions of unpaired electrons with nitrogen, hydrogen in β-position, and hydrogen in γ-position. If no interactions were present, we would observe the 1:1:1:1 quartet. The splitting of the adduct can be attributed to the DMPO-OCH_3_ adduct formed by the reaction with the solvent (DMSO) under visible-light illumination [86], or due to the interactions of O_2_ with DMPO-${\;O}_{2}^{-}\cdot$ [87]. Again, the CNTR/2 photocatalyst produced more of DMPO-${\;O}_{2}^{-}\cdot$ adduct compared to the CNTP/2 sample, which can be attributed to the enhanced presence of more reductive *e*^−^ in the CB of CN due to the partial Z-scheme mechanism with the Ti_x_(^3+^)O_2-x_ part of the TiO_2_ particle in the composite material. Therefore, we can confirm with both scavenging experiments and in situ quenching measurements that, in our system (CNTR/2), both type-II and Z-scheme mechanisms take place, which is the reason for the increased BPA degradation in the case of CNTR/2 nanosolids.

## 3. Experimental Procedure

### 3.1. Synthesis of Materials

All chemicals were of analytical grade and were used without further processing. Ultrapure water (18.2 MΩcm, Millipore, Burlington, MA, USA) was used in all cases. A commercially available g-C_3_N_4_ (TCI, TCI Chemicals, Tokyo, Japan, purity > 95%) was used as a reference for the photocatalytic degradation experiments. Dicyandiamide (DCDA, Sigma Aldrich, Germany, purity > 99%) and a simple thermal polymerization (T_end_ = 550 °C, T_ramp_ = 300 °C/h, t = 4 h) in air were used to prepare g-C_3_N_4_ (CN), as shown in Scheme S1 in the Supplementary Information. A hydrothermal approach in 10 M sodium hydroxide (NaOH, Sigma Aldrich, Darmstadt, Germany, purity > 98%) was used to synthesize TiO_2_ as either aTT (poorly crystalline nanotubes) or TR (single crystalline nanorods) from the commercially available TiO_2_ (DT-51, denoted TP, CristalACTiV*™*. Cristal France SAS, Thann, France). Specifically, 2 g of TP was dispersed in 10 M NaOH (Sigma Aldrich, Germany, purity > 98%) in a 200 mL Teflon-lined autoclave. The hydrothermal synthesis was carried out under stirring at 130 °C for 24 h. After cooling, the resulting product was washed several times to achieve a neutral pH and dispraised in 0.1 M hydrochloric acid (HCl, Sigma Aldrich, Germany, >37%) for the first three washing days and then in ultrapure water for the last two washing days. The product obtained was cryogenically dried for 24 h and labelled as aTT. For the preparation of TR, aTT was used as a precursor, which was heated in aluminum oxide crucibles for 2 h in a muffle furnace at 500 °C (T_ramp_ = 150 °C/h) in air. The g-C_3_N_4_/TiO_2_ composites were synthesized using a mortar mixing technique and a 1:1 ratio of g-C_3_N_4_ to TiO_2_ (50 wt.% g-C_3_N_4_). The obtained photocatalysts were designated as CNTP/M, CNaTT/M and CNTR/M for TP-, and aTT- and TR-containing samples, respectively. In addition, the photocatalysts synthesized in the mortar were annealed in a muffle furnace at 350 °C (T_ramp_ = 300 °C/h) for 2 h in air. The treated photocatalysts were designated as CNTP/2, CNaTT/2 and CNTR/2.

### 3.2. Structural and Textural Properties of the Materials

The Fourier transform infrared spectroscopy (FTIR) spectra of the analyzed materials were recorded using a Perkin-Elmer FTIR Frontier spectrometer equipped with a GladiATR VisionTM accessory from PIKE Technologies or a Perkin Elmer pellet sample holder. All spectra were recorded from 4000 to 450 cm^−1^ with a resolution of 4 cm^−1^ (average of 32 spectra). To prepare a pellet, 1 mg of a sample was ground with 199 mg KBr (Sigma Aldrich, Germany, for IR spectroscopy Uvasol^®^). The sample/KBr mixture was placed in a 13 mm pellet holder and pressed at 5 tons for 5 min.

Phase composition and purity of the produced materials were analyzed using a PANanalytical PRO MPD X-ray diffractometer (XRD) with Cu Kα1 radiation (1.54056 Å) in the scan range between 10 and 90° in 0.034° steps. The HighScore Plus (Malvern Panalytical, Almelo, The Netherlands) was used for sample analysis. The PDFs of the standards were obtained from the International Centre for Diffraction Data (PDF-4+ 2023).

The porosity of the samples was analyzed using nitrogen physisorption analysis using the Micromeritics TriStar II 3020 instrument. Degassing of the materials at 90 °C (60 min) and 180 °C (240 min) was performed with a nitrogen stream (Linde, Dublin, Germany, purity 6.0). Liquid nitrogen was used to cool the samples to −196 °C. The theory of Brunauer, Emmett, and Teller (BET) was used to determine the specific surface area of the photocatalysts, while the theory of Barrett, Joyner, and Halenda (BJH) was used to determine the pore size distribution.

The chemistry and phase composition of the samples were analyzed using a transmission electron microscope (TEM, JEM-2100, JEOL Inc., Peabody, MA, USA) at 200 kV. The pulverized samples were dispersed in EtOH (Sigma Aldrich, Germany, abs.), sonicated for 30 s to prevent agglomeration, and transferred to commercially available TEM grids of amorphous carbon with Ni support. Microscopic images were recorded with a slow-scan CCD camera (Orius SC1000, Gatan Inc., Pleasanton, CA, USA). Morphological analysis of the prepared materials was performed using a field emission scanning electron microscope (FE-SEM) from Carl Zeiss (Oberkochen, Germany), model SUPRA 35 VP, operated at 1 kV.

The temperature-programmed pyridine desorption profiles (pyridine TPD) of the analyzed samples were obtained by heating the sample in an Ar/He stream (20 mL/min) from room temperature to 125 °C (10 °C/min) in a Micromeritics AutoChem II 2920 apparatus. After 10 min, the samples were cooled to 120 °C, whereupon the gas flow was switched to Ar (20 mL/min). Pyridine was added to the sample by dosing pyridine (80 vol%)/Ar into the Ar stream (20 mL/min) flowing through the sample at 120 °C. The desorption of pyridine from the sample into the Ar stream (20 mL/min) was monitored from 120 to 360 °C (heating ramp 10 °C/min).

Zeta potential measurements were performed using a Malvern Panalytical Zetasizer Ultra Red equipped with a Malvern MPT-3 multipurpose titrator. Aqueous hydrochloric acid (HCl, Sigma Aldrich, Germany, 0.25 and 0.025 M) and sodium hydroxide (NaOH, Sigma Aldrich, Germany, 0.25 M) were used to adjust the pH in the measurement area, and a nitrogen stream (Linde, Germany, purity 5.0) was used to purge the samples at a rate of 50 mL/min. For the measurement of zeta potential, 25 mg of a photocatalyst was dispersed in 100 mL ultrapure water for 30 min (400 rpm). The measurements were carried out in duplicate.

### 3.3. Analysis of the Optical and Electronic Properties of Materials

To obtain the UV-Vis spectra of the analyzed materials, we used the Lambda 650 UV-Vis spectrophotometer (Perkin Elmer, Waltham, MA, USA). Background correction was performed with Spectralon©. All measurements were performed in absorption mode from 700 to 300 nm, with a scan rate of 240 nm/min and a slit width of 2.0 nm. The optical band gaps of the analyzed solids were estimated using the Kubelka–Munk theory as follows:

(1) $${\left(\alpha hv\right)}^{2}=A\left(hv-E_{g}^{opt}\right)$$

where α, *hv*, $E_{g}^{opt}$, and *A* are absorption coefficient, photon energy, direct (optical) band gap, and proportionality constant.

The measurements of solid-state photoluminescence (PL) were carried out using the LS-55 fluorescence spectrometer (Perkin Elmer), which is equipped with a fixed piston plate and a powder sample holder. The excitation wavelength for the samples containing g-C_3_N_4_ was 320 nm with a scan rate of 200 nm/min (from 350 to 620 nm) and an emission slit of 2.6 nm. For the TiO_2_ samples, the excitation wavelength was 300 nm with a scan rate of 150 nm/min (from 340 to 560 nm) and an emission slit of 5.5 nm.

Electrochemical impedance spectroscopy (EIS) was performed using a Methrom Autolab potentiostat/galvanostat (model PGSTAT302N) equipped with a FRA32M EIS module and using 0.1 M KOH (Sigma Aldrich, Germany, purity 99%) as electrolyte. The catalyst samples were applied to the surface of the working electrode by dropping 10 μL of the catalyst/absolute ethanol (10 mg of catalyst dispersed in 1 mL of absolute ethanol) suspension onto the carbon working electrode of the Metrohm DropSens disposable screen-printed carbon electrode (model DRP-150). The corresponding experiments were carried out in an electrochemical PTFE cell (Metrohm DropSens, model Ramancell). The EIS spectra of the analyzed catalysts were recorded in a frequency range of 0.1–10^6^ Hz.

An Adani X-band CMS8400 EPR spectrometer was used to obtain solid-state electron paramagnetic resonance (EPR) spectra of the analyzed materials at room and liquid nitrogen temperature. Mg(II)/MgO powder was used as a standard to determine the uncertainty of the measurements of ±0.0005. The powder samples were placed in a quartz sample tube and inserted into the EPR spectrometer (9.4 MHz microwave frequency). For the pure TiO_2_ samples measured at room temperature, the modulation amplitude was 450 µT with a power attenuation of 15 dB and a gain value of 3 × 10^3^ for all measurements. For the TiO_2_ samples at liquid nitrogen temperature, the modulation amplitude was 450 µT with a power attenuation of 15 dB and a gain value of 3 × 10^3^ for all measurements, except for the TP sample, where the gain value was 1 × 103. For samples containing g-C_3_N_4_ at room temperature, the measurements were performed at 338.00 mT (sweep width 20 mT) with a mod. amplitude of 350 µT and a power attenuation of 18 dB (gain value of 8 × 10^2^). For one spectrum, 120 s with three consecutive measurements were used to obtain an average value. An LED light source for visible-light (Schott, Mainz, Germany, model KL 2500; energy spectra can be found in [36]) was used for all illuminations.

### 3.4. XPS Analysis

The XPS measurements were performed using a Supra+ device (Kratos, Manchester, UK). The primary X-ray beam was Al Kα, and the analysis was performed using a 300 × 700 µm spot. The XPS spectra were recorded at pass energies of 160 and 20 eV to obtain overview and high-resolution spectra, respectively. The acceptance angle during the measurements was 90°. The powder samples were fixed on the carbon tape, which was attached to the Si wafer. The base pressure in the spectrometer was 2–10^−9^ Torr. The charge neutralizer was switched on during the measurements. The binding energy (BE) was corrected using a C-C/C-H peak at 284.8 eV in the C 1s spectra, except for sample CN, where the binding energy scale was corrected using a peak representing C in g-C_3_N_4_ at 288.2 eV. Spectra acquisition and processing was performed with ESCApe 1.5 (Kratos).

### 3.5. Tests of the Photocatalytic Activity and Mechanistic Studies of the Prepared Materials

The photocatalytic experiments were carried out in a batch slurry reactor (Lenz Laborglas, Wertheim, Germany) with a 150 W halogen lamp (Philips, Amsterdam, The Netherlands; energy spectra can be found in [36]) equipped with a cut-off filter at 410 nm. Bisphenol A (BPA, Aldrich, purity ≥ 99.0%) was used as model organic pollutants (c_0_ = 10 mg/L, 0.0438 mM). A total of 31.25 mg (c_cat_ = 125 mg/L) of a photocatalyst was suspended in 250 mL of an aqueous BPA solution. The suspension was stirred at 600 rpm and purged with 45 L/h of air. The reaction mixture was thermostatted to 20 °C using a Julabo thermostat (model F25/ME). To establish adsorption/desorption equilibrium, the 10 suspension was kept in the dark for 30 min (gray area in the photocatalytic diagrams). During illumination with visible light, samples were taken at certain times of the reaction and filtered using a 0.2 µm membrane made of regenerated cellulose. For the reactive species quenching experiments, we used p-benzoquinone (BQ, Sigma Aldrich, Germany, purity > 99%), sodium azide (NaN_3_, Merck, Rahway, NJ, USA), methanol (MeOH, Sigma Aldrich, Germany, purity >99%) or tert-butyl alcohol (t-BuOH, Sigma Aldrich, Germany, purity >99%), and formic acid (HCOOH, Kemika, purity >99%) as traps for $O_{2}^{-}\cdot$, ^1^O_2_, OH· and *h^+^*, respectively. The concentration of the traps was 10 mM in all cases, using the same experimental setup as for the photooxidation of BPA.

An HPLC LC-40 (Shimadzu, Kyoto, Japan) with a 100 × 4.6 mm BDS Hypersil C18 (2.4 µm) column was used to analyze the time-dependent BPA concentration. The mobile phase was a mixture of methanol (Merck) and ultrapure water (volume ratio 70:30) with a flow rate of 0.5 mL/min. The column was thermostatted to 30 °C and the autosampler to 25 °C. Detection was carried out with a PDA detector at 190–350 nm and a cell temperature of 40 °C. The determination of total organic carbon (TOC) in fresh and treated BPA solutions was carried out using a Shimadzu TOC-L analyzer equipped with an ASI-L autosampler. The Perkin Elmer CHNS 2400 Series II analyses was used to determine the extent of carbon deposition on the catalyst samples after photocatalytic oxidation of BPA and to calculate the extent of actual mineralization of the organic precursors.

Using a photoluminescence method and the probe molecule coumarin (Alfa Aesar, purity 98%), we investigated the tendency to form hydroxyl radicals (OH·). A total of 10 mg of an investigated photocatalyst was suspended in a 50 mL aqueous solution of coumarin (1.4 mM), stirred at 400 rpm and kept in the dark for 30 min to establish the sorption equilibrium. A Schott KL 2500 LED lamp with a UV cut-off filter at 410 nm was used as a source of visible light (energy spectra can be found in [36]). Samples of the liquid phase were taken at different times during the reaction and filtered using a 0.2 µm filter made of regenerated cellulose membrane. A total of 300 µL of the filtered solution was diluted with ultrapure water in a 10 mL flask and measured using a Perkin Elmer LS-55 fluorescence spectrometer. The excitation wavelength was 338 nm at a scan rate of 200 nm/min (from 200 to 600 nm) and the emission slit was set to 10 nm. The concentration of 7-hydroxycoumarin was calculated using the calibration curve from [31].

Spin trapping experiments were performed using 5-5-dimethyl-1-pyrroline-N-oxide (DMPO, Sigma Aldrich, purity ≥ 98.0%) dissolved in dimethyl sulfoxide (DMSO, Sigma Aldrich, purity ≥ 99.9%) as solvent. The measurements of the corresponding spin adduct (DMPO-$O_{2}^{-}\cdot$) were performed using a 100 μL liquid flat cell (Fluorochem, model WG-808_Q) and an Adani CMS8400 EPR spectrometer. The illumination source for the visible light was the Schott KL 2500 LED lamp (energy spectra can be found in [36]). The DMPO experiments were performed at 337.00 mT (sweep width 10 mT) with a mod. amplitude of 200 µT and a power attenuation of 15 dB (gain value of 4 × 10^3^). The initial concentration of DMPO was 4 g/L and the concentration of the catalyst was 2 g/L.

## 4. Conclusions

The aim of the study was to evaluate the photocatalytic performance of g-C_3_N_4_/TiO_2_ composites with different TiO_2_ morphologies. Poorly crystalline nanotubes (aTT, S_BET_ = 336 m^2^/g) and crystalline anatase nanorods (TR, S_BET_ = 100 m^2^/g) were synthesized from commercial TiO_2_ (TP, S_BET._= 82 m^2^/g) and incorporated into composites with g-C_3_N_4_ (CN, S_BET_ = 17 m^2^/g) via mortar (M) or 2 h calcination (2). All composites confirmed the presence of g-C_3_N_4_ and TiO_2_ with a preserved mesoporous structure. SEM and TEM analyses showed the ellipsoidal shape of TP, aTT as quasi-nanotubes, and TR as elongated rods. UV-Vis DR spectra showed UV absorption for TiO_2_ and visible-light absorption for CN, with CNTR composites showing the highest visible-light absorption. PL and EIS showed improved the carrier dynamics (the R_CT_ of bare TiO_2_ and CN was between 0.84 MΩ and 1.0 MΩ), especially for CNTP/2 (R_CT_ = 0.48 MΩ) and CNTR/2 (R_CT_ = 0.40 MΩ), with the latter performing best. Ti^3+^ and O vacancies improved visible-light absorption and charge separation. Furthermore, the EPR and XPS measurements indicated that Ti^3+^ was mainly present at the surface in the case of the TR sample, implying (i) a higher visible-light absorption of TiO_2_ and (ii) probably a higher tendency to form a close interface with the CN component. The degradation of BPA under visible-light illumination showed increased rates for all composites compared to pure TiO_2_ (between 2.4 and 4% BPA degradation and TOC_M_ between 0.6 and 2.4% after 2 h illumination) or CN (12% BPA degradation and TOC_M_ of 7.8% after 2 h illumination), with CNTR/2 (30% BPA degradation and TOC_M_ of 25.4% after 2 h illumination) achieving the highest activity due to improved visible light utilization and charge separation. In addition, the presence of Ti^3+^ enabled the partial occurrence of the Z-scheme mechanism, whereby the CNTR/2 photocatalyst exhibited improved redox properties. However, this mechanism was limited due to the still low amount of Ti^3+^ in the TR component (limited area of TiO_2_ particle), so a dual mixed type-II/Z-scheme mechanism probably occurred. Since the type-II mechanism was still the most pronounced, this limits the actual photocatalytic activity of the nanosolid. This study highlights the importance of the synthesis method in influencing the photocatalytic behavior, beyond the morphology, and emphasizes the potential of these composites for environmental remediation. A future pathway to increase the catalytic activity of the CNTP/2 catalyst includes several strategies, such as increasing the Ti^3+^ concentration in the TiO_2_ component, optimizing the g-C_3_N_4_/TiO_2_ ratio, applying longer calcination times, doping with elements such as nitrogen, sulfur, or plasmonic metals, and optimizing the reaction conditions, etc. These approaches aim to improve the charge transfer and maximize photocatalytic activity for environmental remediation.

## Figures and Tables

**Table 1:** Results of nitrogen physisorption experiments (specific surface area (S_BET_), pore volume (V_pore_), and pore diameter (d_pore_)), estimated crystallite size for TiO_2_ particles at 48° 2θ calculated using the Scherrer equation for spherical particles using the results of the XRD analysis, and peak positions of the (002) peak for g-C_3_N_4_ materials (CN). In all cases, the standard deviation was rounded up to the corresponding trustworthy decimal place.

Sample	S_BET_	V_pore_	d_pore_	Crystallite Size @ 48° 2θ	CN peak Position of (002)
	m^2^/g	cm^3^/g	nm	nm	° 2θ
CN	17 ± 1	0.08 ± 0.01	20.4 ± 0.02	/	27.661
TP	82 ± 1	0.29 ± 0.01	13.7 ± 0.01	18.6 ± 0.1	/
CNTP/M	57 ± 1	0.23 ± 0.02	16.8 ± 0.03	22.4 ± 0.2	27.660
CNTP/2	50 ± 1	0.21 ± 0.01	15.9 ± 0.01	22.4 ± 0.1	27.595
aTT	337 ± 6	0.99 ± 0.04	11.7 ± 0.04	/	/
CNaTT/M	164 ± 3	0.51 ± 0.02	12.6 ± 0.02	/	27.529
CNaTT/2	163 ± 3	0.50 ± 0.01	12.4 ± 0.01	13.7 ± 0.5	27.463
TR	100 ± 2	0.47 ± 0.02	18.9 ± 0.03	22.4 ± 0.1	/
CNTR/M	57 ± 1	0.26 ± 0.01	18.2 ± 0.01	22.4 ± 0.2	27.562
CNTR/2	53 ± 1	0.24 ± 0.01	18.3 ± 0.02	22.0 ± 0.1	27.496

**Table 2:** The reported values of total organic carbon (TOC) removal (which represent a sum of TOC mineralization (TOC_M_) and TOC accumulation (TOC_A_)) and corresponding values of BPA degradation measured at the end of the BPA oxidation runs. In all cases, the standard deviation is rounded up to the corresponding trustworthy decimal place.

Sample	^a^ TOC_removal_	^a^ TOC_M_	^a^ TOC_A_	BPA Degradation
	%			
CN	8.6	7.8	0.8	11.4 ± 0.2
TP	0.8	0.7	0.2	2.4 ± 0.0
CNTP/M	10.1	8.7	1.4	11.8 ± 0.2
CNTP/2	13.2	11.3	1.9	14.3 ± 0.2
aTT	1.1	0.6	0.5	2.5 ± 0.0
CNaTT/M	15.5	14.1	1.4	16.5 ± 0.2
CNaTT/2	25.1	22.9	2.2	26.3 ± 0.2
TR	2.6	2.4	0.2	4.0 ± 0.0
CNTR/M	24.9	23.6	1.3	26.0 ± 0.1
CNTR/2	27.1	25.4	1.7	30.3 ± 0.1

^a^ The standard deviation for all TOC measurements did not exceed ±0.1.

**Table 3:** Comparison of the photocatalytic degradation performance of the synthesized photocatalysts with previously reported photocatalysts from the literature for BPA removal (BPA degr.) after 120 min of light exposure.

Photocatalyst	Catalysts Dosage	C_0_(BPA)	BPA Degr.	Light Source	Ref.
	mg/L	mg/L	%		
g-C_3_N_4_/TiO_2_ *	0.125	10	21.5	150 W, Halogen lamp	[36]
TiO_2_ **	0.125	10	9.0		
g-C_3_N_4_	0.125	10	11.4		
g-C_3_N_4_/TiO_2_	0.5	20	38.0	sunlight	[71]
TiO_2_	0.5	20	30.0		
g-C_3_N_4_	0.5	20	25.0		
g-C_3_N_4_/TiO_2_ *	0.5	10	25.0	200 W, LED Flood light	[72]
TiO_2_ ***	0.5	10	25.0		
g-C_3_N_4_/TiO_2_ *	0.125	10	30.3	150 W, Halogen lamp	This study
TiO_2_	0.125	10	4.0		
g-C_3_N_4_	0.125	10	11.4		

* Ratio between g-C_3_N_4_ and TiO_2_ in the composite is 1:1 ** Commercially available TiO_2_ (XT25376, Saint-Gobain, Merrimack, NH, USA). *** N doped TiO_2_.

## Data Availability

Data will be made available on request.

## Supplementary Materials

The following supporting information can be downloaded at: https://www.mdpi.com/article/10.3390/molecules30030460/s1, Scheme S1. Graphical representation of the synthesis process with estimated yields.; Figure S1. (a) Nitrogen physisorption isotherms and (b) corresponding BJH pore size distributions of the investigated photocatalysts.; Figure S2. Pyr-TPD profiles of the analysed (a) TiO_2_ morphologies and CN components, and (b) TiO_2_/g-C_3_N_4_ composites.; Figure S3. Results of zeta potential measurements as a function of pH for (a) CNTP, (b) CNaTT and (c) CNTR series; (d) comparison of composites annealed for 2 h.; Figure S4. SEM micrographs of (a) pure CN, (b) CNTP/2, (c) CNaTT/2 and (d) CNTR/2 samples.; Figure S5. (a) HR-TEM micrograph of a TiO_2_ crystal with an FFT pattern indexed for anatase TiO_2_; the pattern derives from the area marked in red. The area was filtered with average-background-subtraction (ABSF), with the lattice planes marked and the crystal structure displayed in the same orientation. From the outline of the particle, a 3D model was proposed based on the crystallographic file of anatase. (b) Similar to the nanorods, the distances between the planes were measured to be 3.25 Å, which corresponds to the (003) plane in the reference PDF X-ray data.; Figure S6. Gaussian deconvolution of the solid-state photoluminescence (PL) spectra of (a) CN, (b) CNTP/2, (c) CNaTT/2 and (d) CNTR/2 samples.; Figure S7. Nyquist plots for (a) CNTP, (b) CNaTT and (c) CNTR series and (d) comparison of TiO_2_/g-C_3_N_4_ composites annealed for 2 h. Figure (e) shows the electrochemical equivalent circuit used to fit the Nyquist plots, which includes a solution resistance (R_S_), a charge transfer resistance (R_CT_), the Warburg impendence (W) and the constant phase element (CPE).; Figure S8. EPR spectra of pure solid TiO_2_ morphologies obtained at room temperature (a) without and (b) with visible-light illumination. The modulation amplitude was 450 µT with a power attenuation of 15 dB and a gain value of 3 × 103 for all measurements.; Figure S9. EPR solid-state spectra of pure TiO_2_ morphologies obtained at liquid nitrogen temperature (LN2). The modulation amplitude was 450 µT with a power attenuation of 15 dB and a gain value of 3×103 for all measurements, except for the TP sample where the gain value was 1 × 103 (Figure S9 contains the corrected TP spectra multiplied to obtain the same gain value for an easier comparison).; Figure S10. Room temperature solid-state EPR spectra for (a) pure CN, (b) TiO_2_/g-C_3_N_4_ mortar and (c) TiO_2_/g-C3N4 series annealed for 2 h.; Figure S11. Fitted high-resolution XPS spectra for (a) C 1s, (b) N 1s, and (c) O 1s in CNTR/2 sample.; Figure S12. (a) Results of BPA degradation with pure components under 2 h of visible-light irradiation and (b) BPA photooxidation with different CNTR/2 concentrations under 6 h of visible-light. The average ± standard deviation of triplicate tests is shown. Figure (c) shows the ATR-FTIR spectra of fresh and reused CNTR/2 photocatalysts (after 3 BPA degradation cycles), with the inset showing the KBr/CNTR/2 transmission FTIR spectra. Figure (d) shows the XRD patterns of the fresh and reused CNTR/2 samples.; Figure S13. (a) Concentration of 7-HOCU after 120 min illumination of the catalyst/coumarin suspension with visible-light and (b) DMPO/DMSO/catalyst results of the liquid-phase EPR measurements for CNTP/2 and CNTR/2 series after 15 min illumination with visible-light.; Table S1: Results of the CHNS elemental analysis of fresh and used photocatalysts. Nitrogen and hydrogen were not detected in the pure TiO_2_ samples. Only carbon and hydrogen were measured in the used photocatalysts. The observed error of three repetitions was within ± 1%.; Table S2: Results of the temperature-programmed pyridine desorption measurements of the analyzed materials to determine the acidic surface sites (AcSS) (moles of adsorbed pyridine from peak area, calculated concentration and density of AcSS).; Table S3: Determined point of zero charge (pHPZC, from pH conditioned zeta-potential measurements in Figure S3), optical band gap value (opt_Eg_, obtained from the UV-Vis DR measurements in Figure 5) and charge transfer resistance (RCT, obtained from the Nyquist plots in Figure S7). The observed error of two repetitions for pH_PZC_ was within ± 1% and for R_CT_ within ± 2%. The observed error of three repetitions for opt_Eg_ was within ± 2%.; Table S4: Peak position and peak area of the transitions obtained by the three peak Gaussian deconvolution of the solid-state PL spectra in Figure S6.
