# Supplementary material for: Effect of TiO2 Morphology on the Properties and Photocatalytic Activity of g-C3N4/TiO2 Nanocomposites Under Visible-Light Illumination
Source: Molecules. 2025 Jan 21;30(3):460. doi: 10.3390/molecules30030460 (PMC11820781; doi:10.3390/molecules30030460)
Supplement: Supplementary file 1 [file molecules-30-00460-s001.zip › molecules-3406631-supplementary.pdf]

Supporting Information

Effect of TiO_2 morphology onto the properties and photocatalytic activity of g-C_3N_4/TiO_2 nanocomposites under visible-light illumination

Matevž Roškarič[a], Gregor Žerjav[a,*], Janez Zavašnik[b], Matjaž Finšgar[c], Albin Pintar[a]

[a]Department of Inorganic Chemistry and Technology, National Institute of Chemistry, Hajdrihova 19, SI-1001 Ljubljana, Slovenia

[b]Gaseous Electronics, Jožef Stefan Institute, Jamova cesta 39, SI-1000 Ljubljana, Slovenia

[c]University of Maribor, Faculty of Chemistry and Chemical Engineering, Smetanova ulica 17, SI-2000 Maribor, Slovenia

[*]Corresponding author. Tel.: +386 1 47 60 249. *E-mail address:* gregor.zerjav@ki.si (G. Žerjav).

1. Results

1.1. Structural and chemical properties

1.1.1 Temperature-programmed pyridine desorption

To observe the surface properties of the investigated photocatalysts, we performed temperature-programmed pyridine desorption (Pyr-TPD) measurements to determine the acidic surface sites (AcSS). It can be seen from Fig. S2a that for the pure components, only TiO_2 shows the presence of AcSS, as the TPD profile for the CN sample is flat. This is also true for the calculated concentration of AcSS, which is practically absent for the CN solid (Table S2). The results are consistent with literature reports indicating that the polymeric g-C_3N_4 material has only a small amount of basic Lewis surface sites [1]. Comparing all pure TiO_2 morphologies, it is found that the TR samples has the highest AcSS concentration (428.0 $\mu mol/g_{cat}$), while the aTT (267.9 $\mu mol/g_{cat}$) and TP (214.7 $\mu mol/g_{cat}$) samples have a similar AcSS concentration (Table S2). Due to the high S_{BET}, the AcSS density in the aTT sample is much lower compared to the TP and TR samples and is similar to that of the CN solid. It is also interesting to note that the maximum of the Pyr-TPD profiles is different for the different TiO_2 morphologies investigated (Fig. S2a). Indeed, the aTT sample exhibits the strongest AcSS, as the Pyr-TPD maximum of desorption exceeds the final desorption temperature and is thus at the highest temperature. Both TP and TR samples exhibit similar AcSS strength, so their effects on catalyst behaviour are likely similar. This information can be beneficial as the AcSS can influence the photocatalytic behaviour [2].

Fig. S2b shows the Pyr-TPD profiles of the TiO_2/g-C_3N_4 composites. It can be seen that all samples have similar AcSS strength, except for the CNaTT/M composite, which has the highest

strength. Since the AcSS content comes from the TiO_2 component, it is not surprising that the CNaTT/M composite has the highest AcSS strength, as the aTT component did not change during the synthesis of the mortar composite. However, when the CNaTT/M composite is annealed for 2 h, its crystallinity improves and becomes more similar to that of the TR sample (Fig. 2, XRD). Thus, the character of the CNaTT/2 composite is more or less similar to that of the CNTR/M material. A comparison of the mortar composites shows that both CNaTT/M and CNTR/M solids have a similar AcSS concentration and that the CNTP/M composite has the lowest content (Table S2). Interestingly, the CNaTT/2 composite has the highest AcSS concentration among the annealed samples (453.9 $\mu mol/g_{cat}$), while the CNTR/2 and CNTP/2 composites have similar values (155.4 and 182.4 $\mu mol/g_{cat}$). However, considering the difference in specific surface area, it is found that the AcSS density of the annealed samples is similar regardless of the TiO_2 component used, so it probably has no significant effect on the photocatalytic activity.

1.1.2. Zeta potential measurements

We also performed pH-related zeta potential measurements (Fig. S3 a-d) to determine the surface charge and the point of zero charge (pH_{PZC}) of the investigated photocatalysts. From Table S3, we can conclude that the pure CN sample has a pH_{PZC} value (3.8) characteristic of g-C_3N_4 materials [3]. Furthermore, the TP and TR samples have values of 5.0 and 5.7, in contrast to the aTT sample, which has the lowest value of 3.7. This is probably due to the poor crystalline character, which affects the dynamics of the surface charge. When the CN component is added, the pH_{PZC} value for the CNaTT/M composite is the same, but a decrease from 5.7 (TR) to 5.3 (CNTR/M) and an increase from 5.0 (TP) to 5.5 (CNTP/M) is observed. In both cases, the values have become similar. When the CNTP/M and CNTR/M composites are annealed to

improve the junction, the pH_{PZC} value decreases in both cases at the same value of 0.4. Due to the lower starting point in the CNTR/M composite, the absolute value of the CNTR/2 material (4.9) is lower than that of the CNTP/2 solid (5.1). We can conclude that both materials have successfully formed a heterojunction and that in the case of the CNTR series the junction could be slightly different due to the lower pH_{PZC} indicating more difficult access to the functional groups. If the components are “fused" together”, access would be limited and we would observe a more drastic decrease in S_{BET}, which was the case for the CNTR series. For the aTT series, the additional annealing of the CNaTT/M composite increases the pH_{PZC} to 4.6 due to crystallization of the aTT component. Since the pH of the aqueous BPA solution is around 6.2, this means that the surface of all photocatalysts is negatively charged and probably does not play a crucial role in the photocatalytic experiments with BPA as a model organic pollutant.

1.2. Morphological analysis

SEM analysis was used to obtain information on the morphology of the solids investigated. The SEM images of the pure CN sample and all annealed TiO_2/g-C_3N_4 composites are shown in Fig. S4. It can be seen that the pure CN sample forms large aggregates of g-C_3N_4 particles (Fig. S4a). In the TiO_2/g-C_3N_4 composites, the CN component acts as a binder for the TiO_2 component. In the case of the CNTP/2 composite (Fig. S4b), the formation of large aggregates of both components can be observed. The TiO_2 nanoparticles (elliptical particles) are probably mainly deposited on the surface of the CN component. In the case of CNaTT/2 and CNTR/2 composites (Figs. S4c and S4d), TiO_2 nanorods intertwined with the CN component are clearly visible. As can be seen in the XRD analysis (Fig. 2), the CNaTT/2 composite exhibits improved crystallinity, indicating the transition of the poorly crystalline nanotubes to nanorod morphology, as shown in Fig. S4c. The intertwining of aTT or TR and CN components could

be more favorable than the deposition of TiO_2 on CN (as in the case of TP and CN), since a complex network in the case of CNTR/2 and CNaTT/2 composites potentially greatly increases the charge carrier mobility.

References

[1] M.R. Rajeshwari, S. Kokilavani, S.S. Khan, Recent developments in architecturing g-C_3N_4 based nanostructured photocatalysts: Synthesis, modifications and applications in water treatment, Chemosphere 291 (2022) 132735. https://doi.org/10.1016/j.chemosphere.2021.132735.

[2] L. Palmisano, V. Augugliaro, M. Schiavello, A. Sclafani, Influence of acid-base properties on photocatalytic and photochemical processes, J. Mol. Catal. 56 (1989) 284-295. https://doi.org/10.1016/0304-5102(89)80192-0.

[3] B. Zhu, P. Xia, W. Ho, J. Yu, Isoelectric point and adsorption activity of porous g-C_3N_4, Appl. Surf. Sci. 344 (2015) 188-195. https://doi.org/10.1016/j.apsusc.2015.03.086.

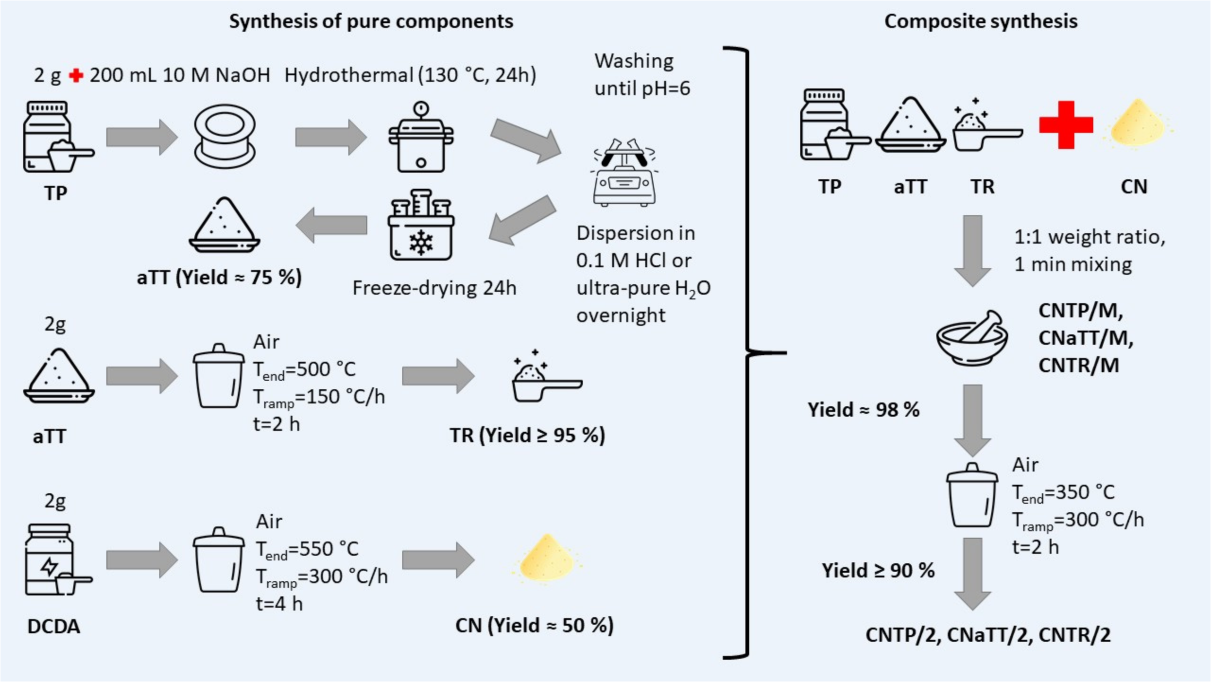

Scheme 1. Graphical representation of the synthesis process with estimated yields.

a)

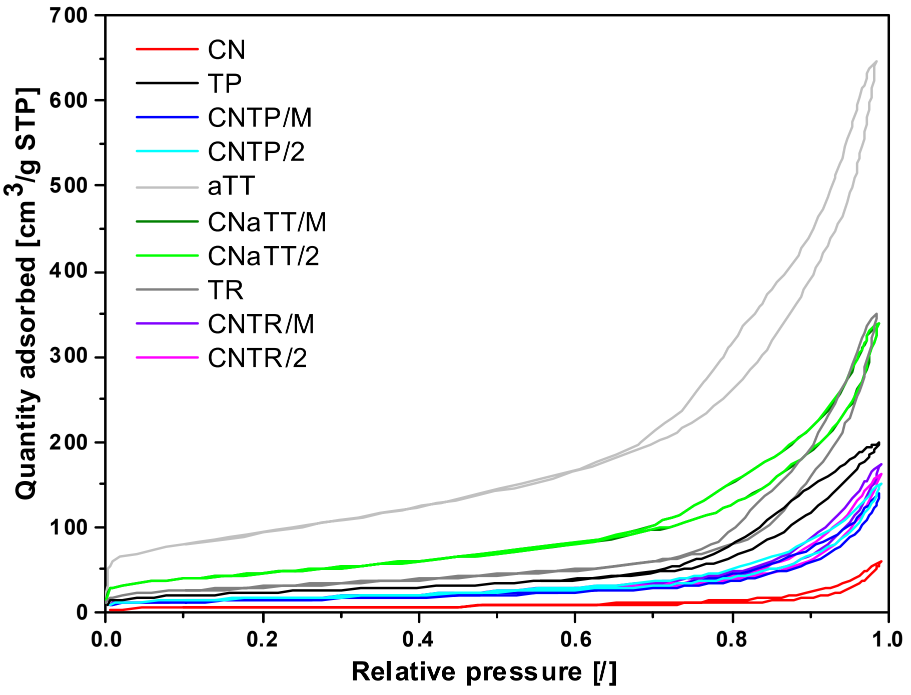

b)

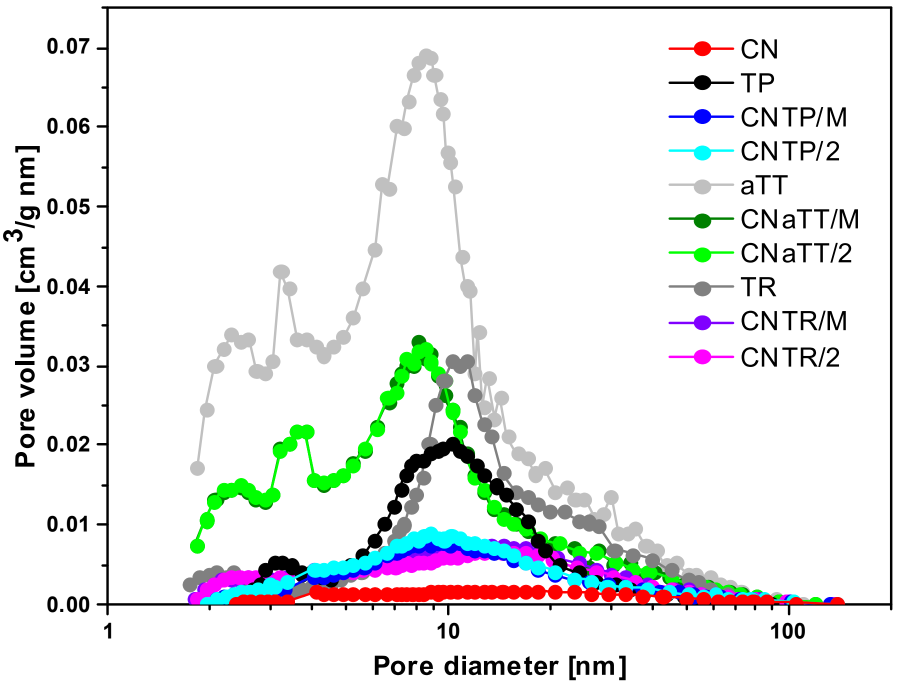

Figure S1. a) Nitrogen physisorption isotherms and b) corresponding BJH pore size distributions of the investigated photocatalysts.

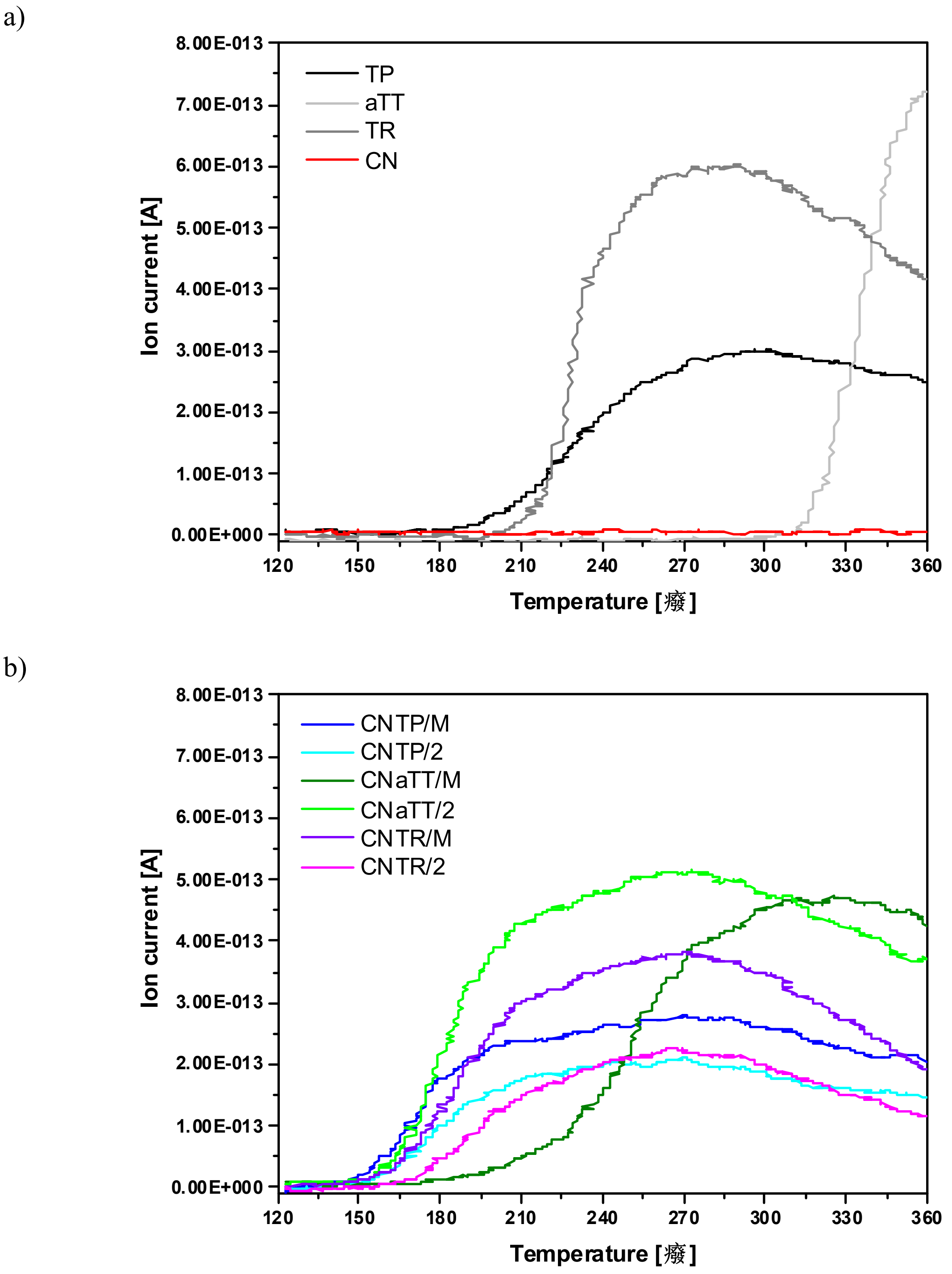

Figure S2. Pyr-TPD profiles of the analysed a) TiO_2 morphologies and CN components, and b) TiO_2/g-C_3N_4 composites.

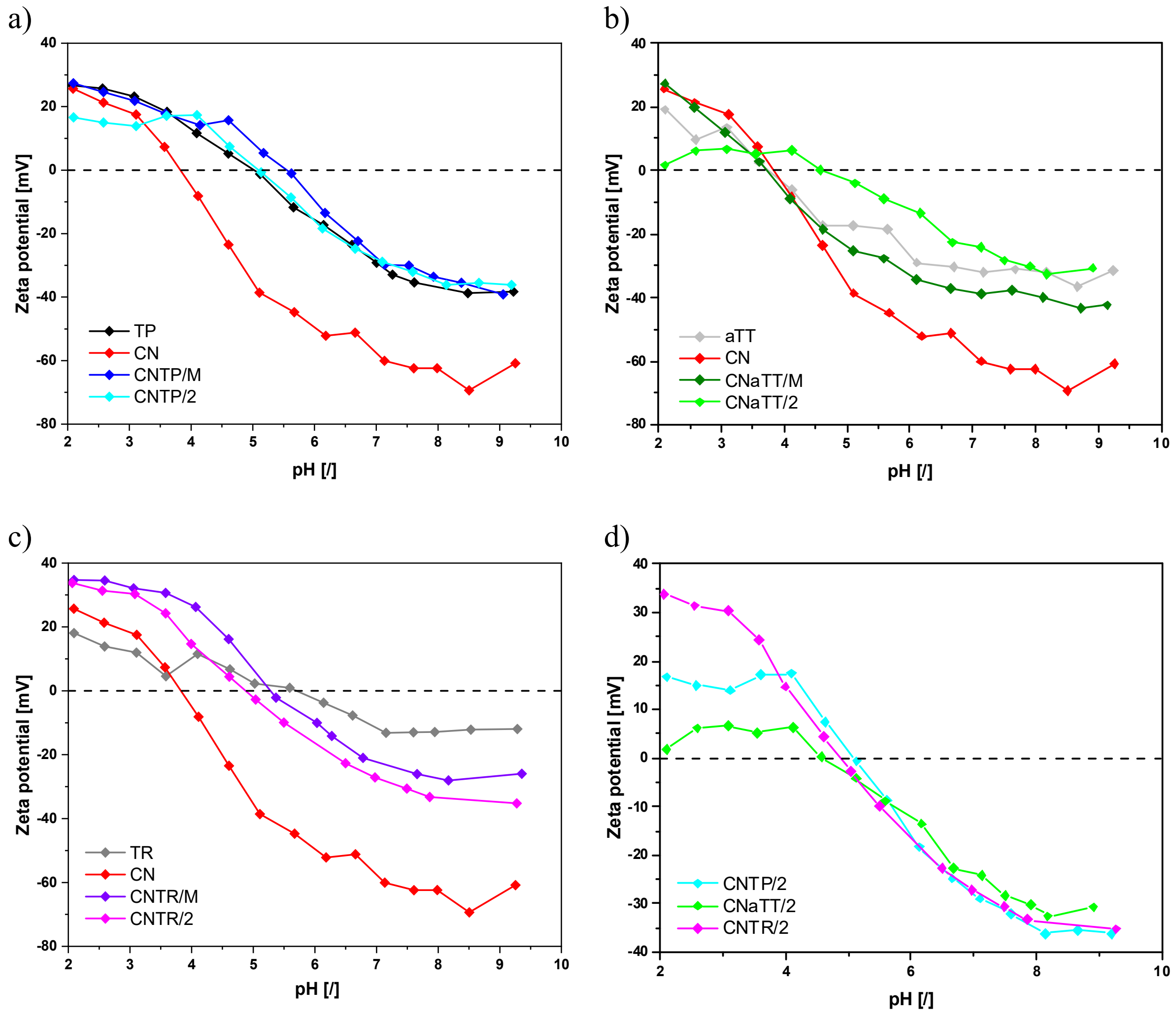

Figure S3. Results of zeta potential measurements as a function of pH for a) CNTP, b) CNaTT and c) CNTR series; d) comparison of composites annealed for 2 h.

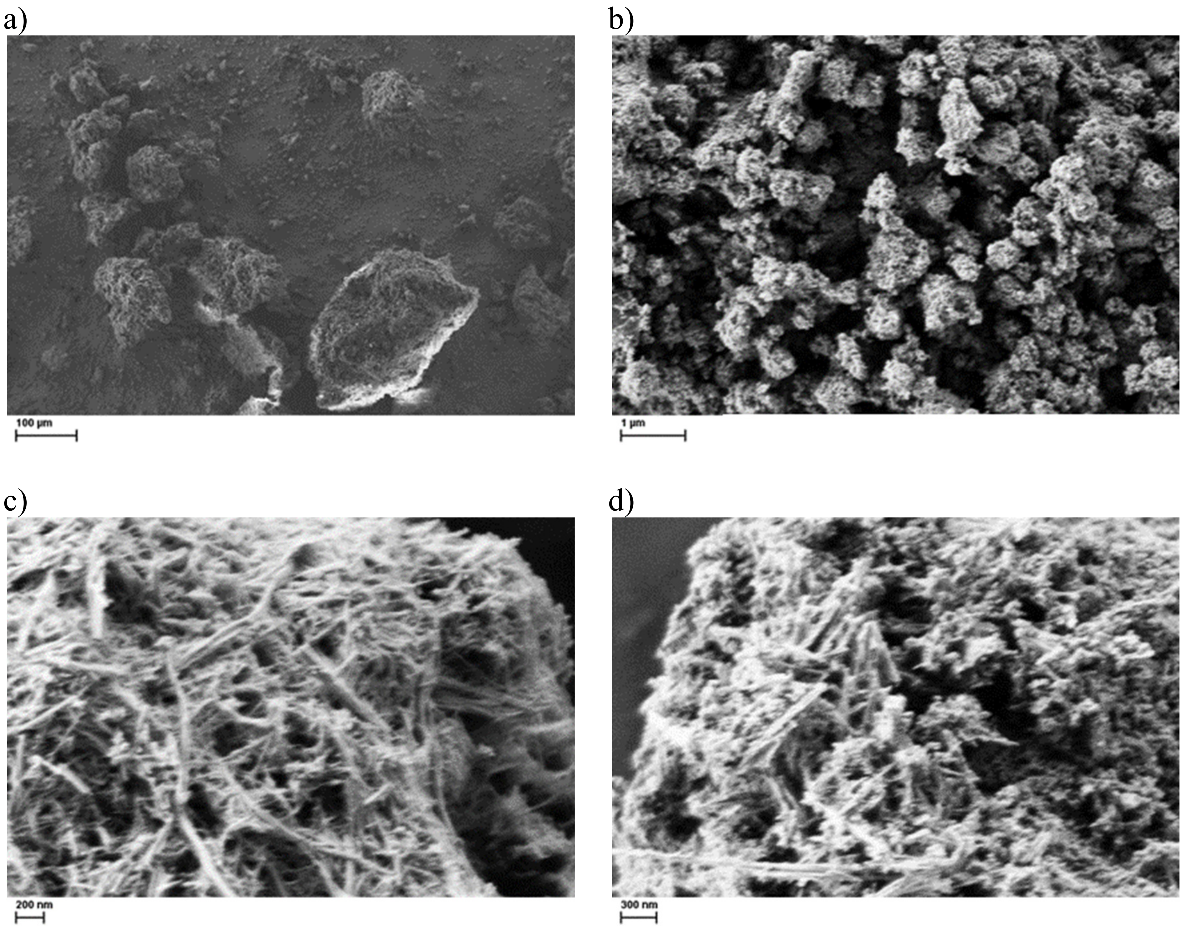

Figure S4. SEM micrographs of a) pure CN, b) CNTP/2, c) CNaTT/2 and d) CNTR/2 samples.

a)

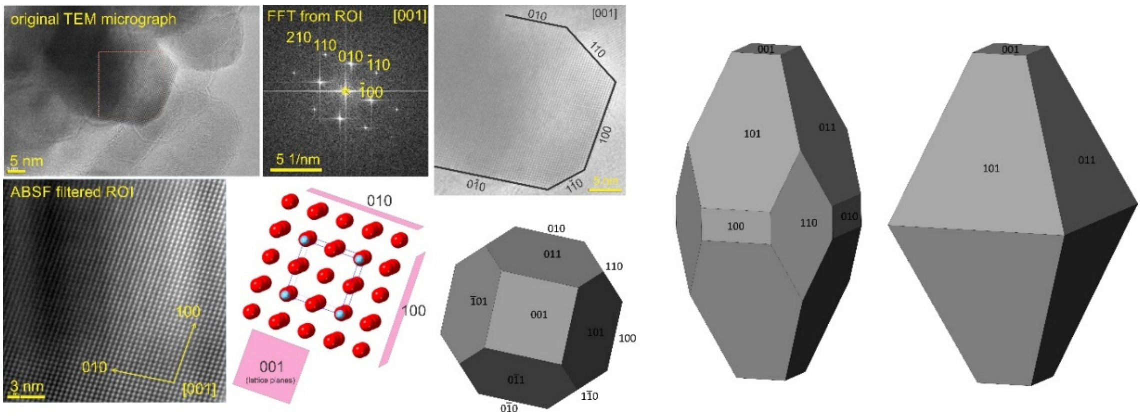

b)

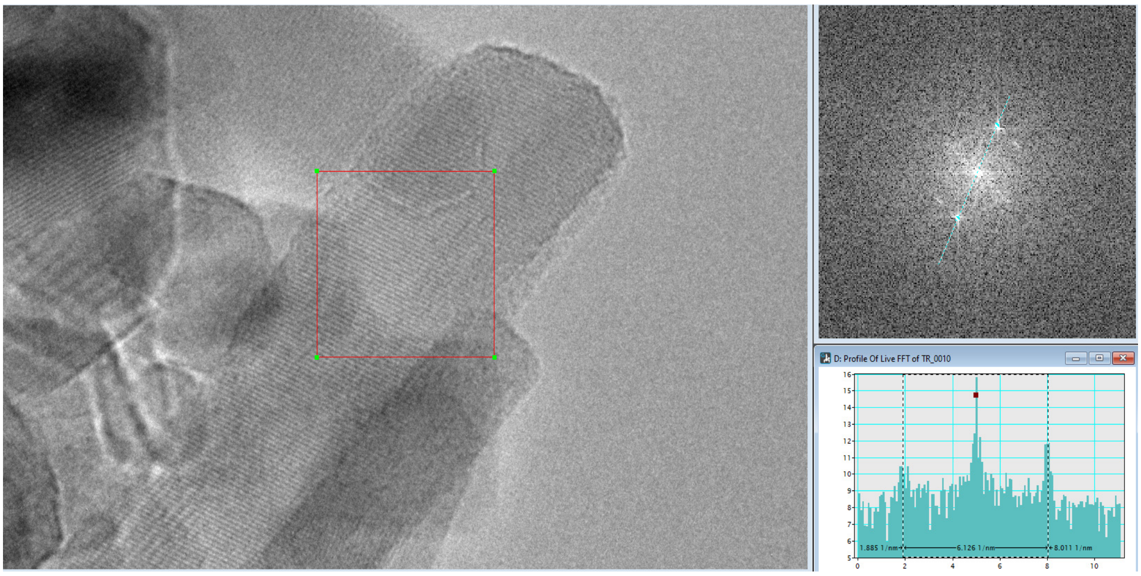

Figure S5. a) HR-TEM micrograph of a TiO_2 crystal with an FFT pattern indexed for anatase TiO_2; the pattern derives from the area marked in red. The area was filtered with average-background-subtraction (ABSF), with the lattice planes marked and the crystal structure displayed in the same orientation. From the outline of the particle, a 3D model was proposed based on the crystallographic file of anatase. b) Similar to the nanorods, the distances between the planes were measured to be 3.25 Å, which corresponds to the (003) plane in the reference PDF X-ray data.

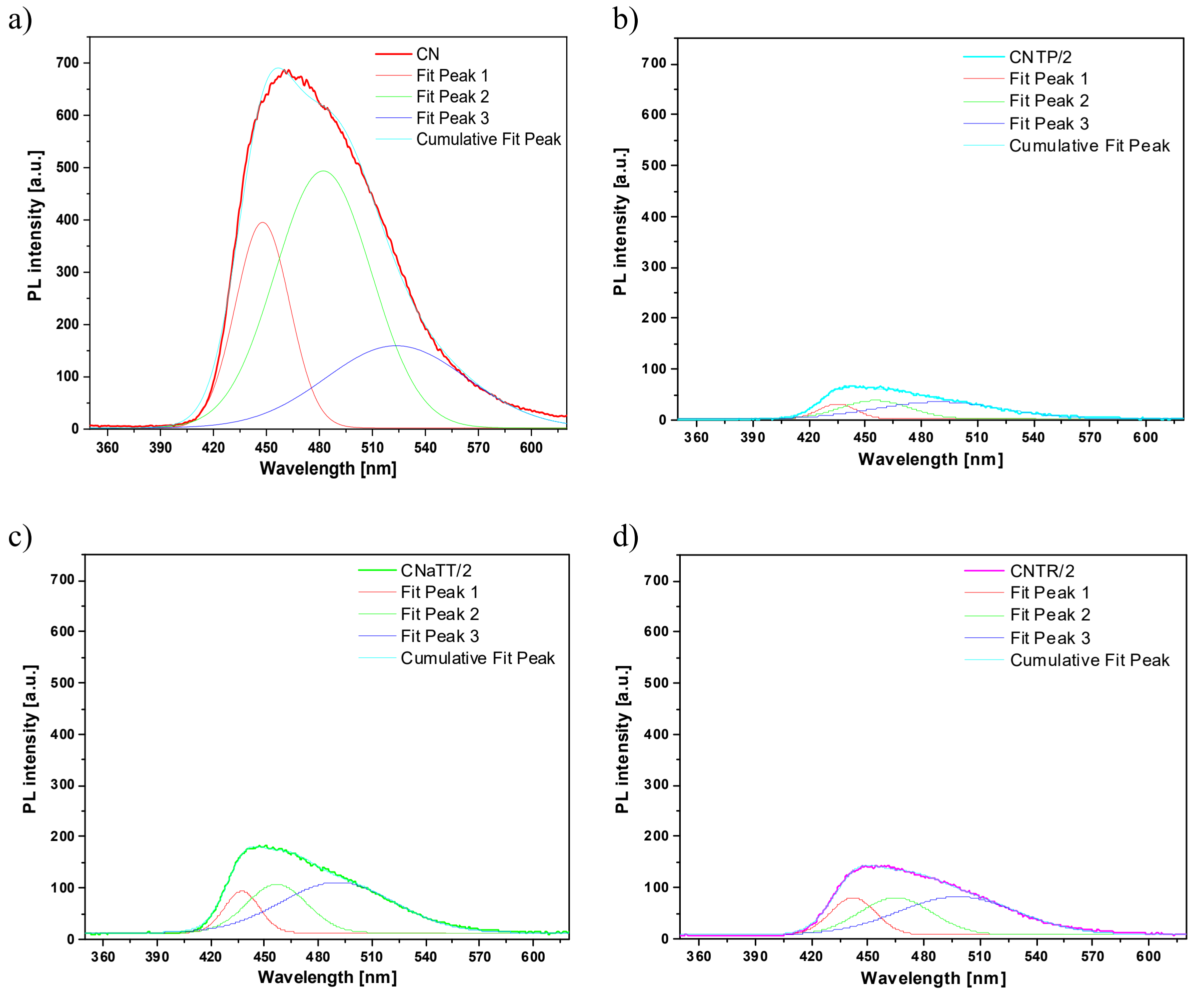

Figure S6. Gaussian deconvolution of the solid-state photoluminescence (PL) spectra of a) CN, b) CNTP/2, c) CNaTT/2 and d) CNTR/2 samples.

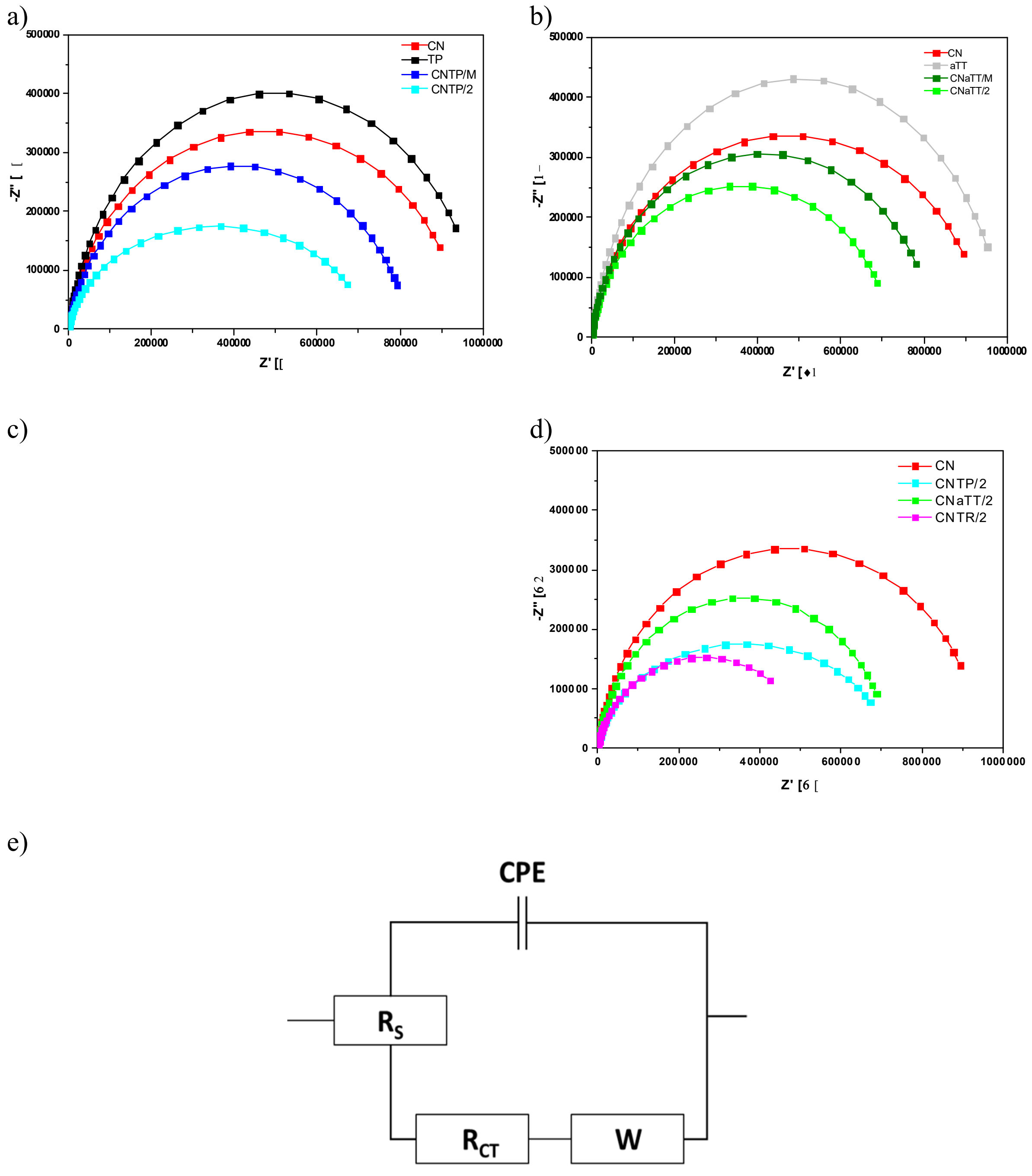

Figure S7. Nyquist plots for a) CNTP, b) CNaTT and c) CNTR series and d) comparison of TiO_2/g-C_3N_4 composites annealed for 2 h. Figure e) shows the electrochemical equivalent circuit used to fit the Nyquist plots, which includes a solution resistance (RS), a charge transfer resistance (R_{CT}), the Warburg impendence (W) and the constant phase element (CPE).

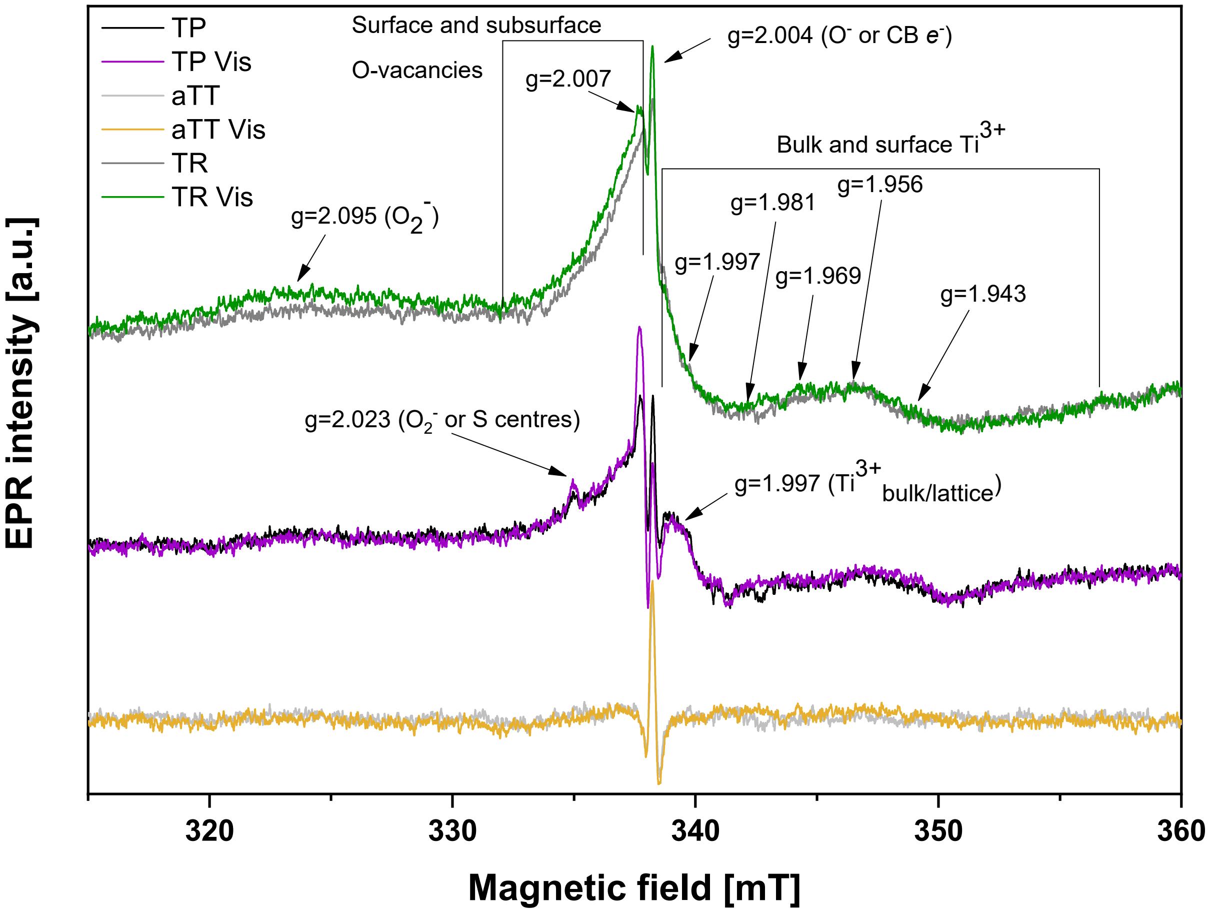

Figure S8. EPR spectra of pure solid TiO_2 morphologies obtained at room temperature a) without and b) with visible-light illumination. The modulation amplitude was 450 µT with a power attenuation of 15 dB and a gain value of 3×10^3 for all measurements.

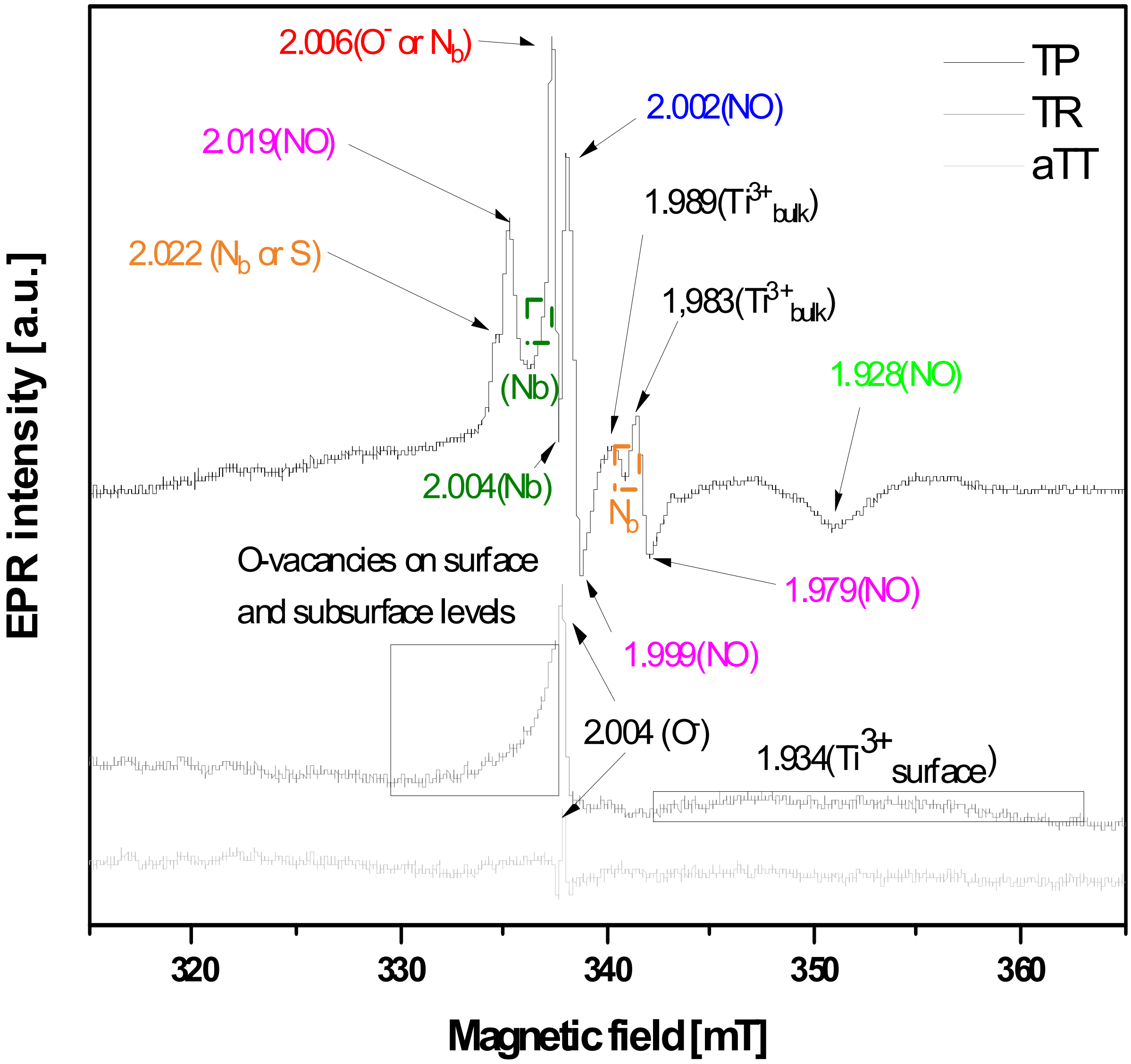

Figure S9. EPR solid-state spectra of pure TiO_2 morphologies obtained at liquid nitrogen temperature (LN2). The modulation amplitude was 450 µT with a power attenuation of 15 dB and a gain value of 3×10^3 for all measurements, except for the TP sample where the gain value was 1×10^3 (Fig. S9 contains the corrected TP spectra multiplied to obtain the same gain value for an easier comparison).

a)

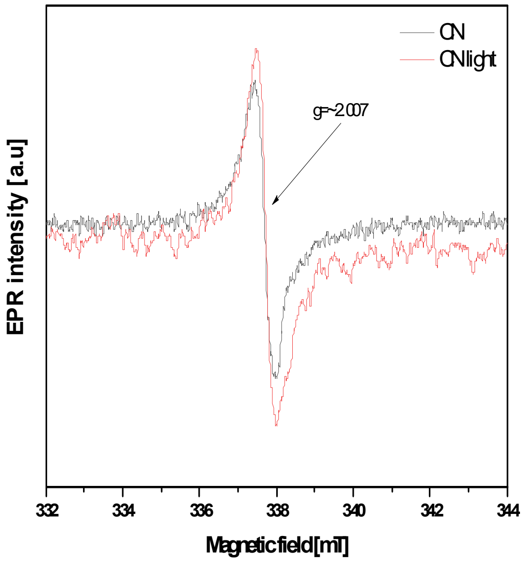

b)

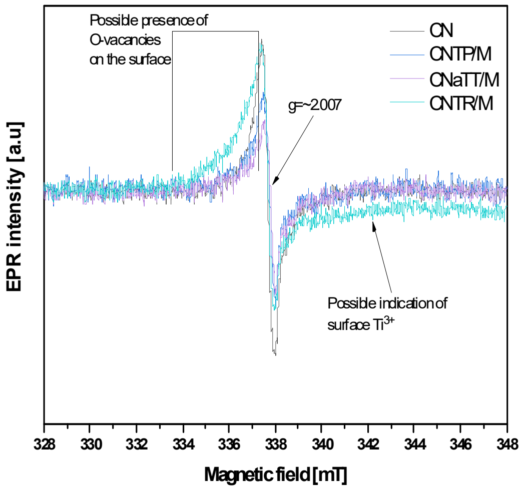

c)

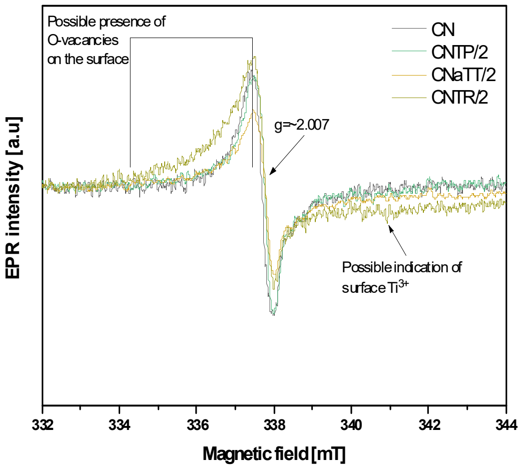

Figure S10. Room temperature solid-state EPR spectra for a) pure CN, b) TiO_2/g-C_3N_4 mortar and c) TiO_2/g-C_3N_4 series annealed for 2 h.

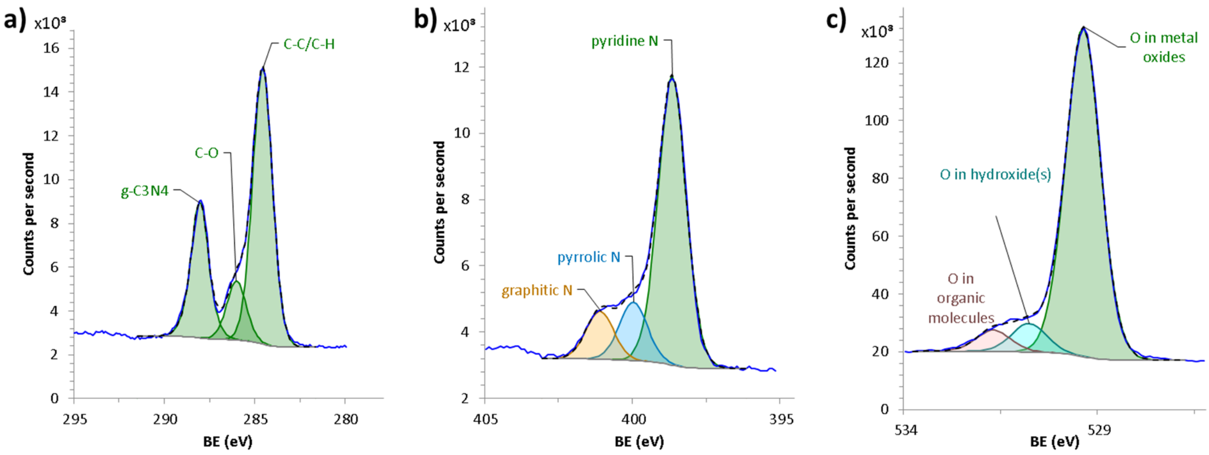

Figure S11. Fitted high-resolution XPS spectra for a) C 1s, b) N 1s, and c) O 1s in CNTR/2 sample.

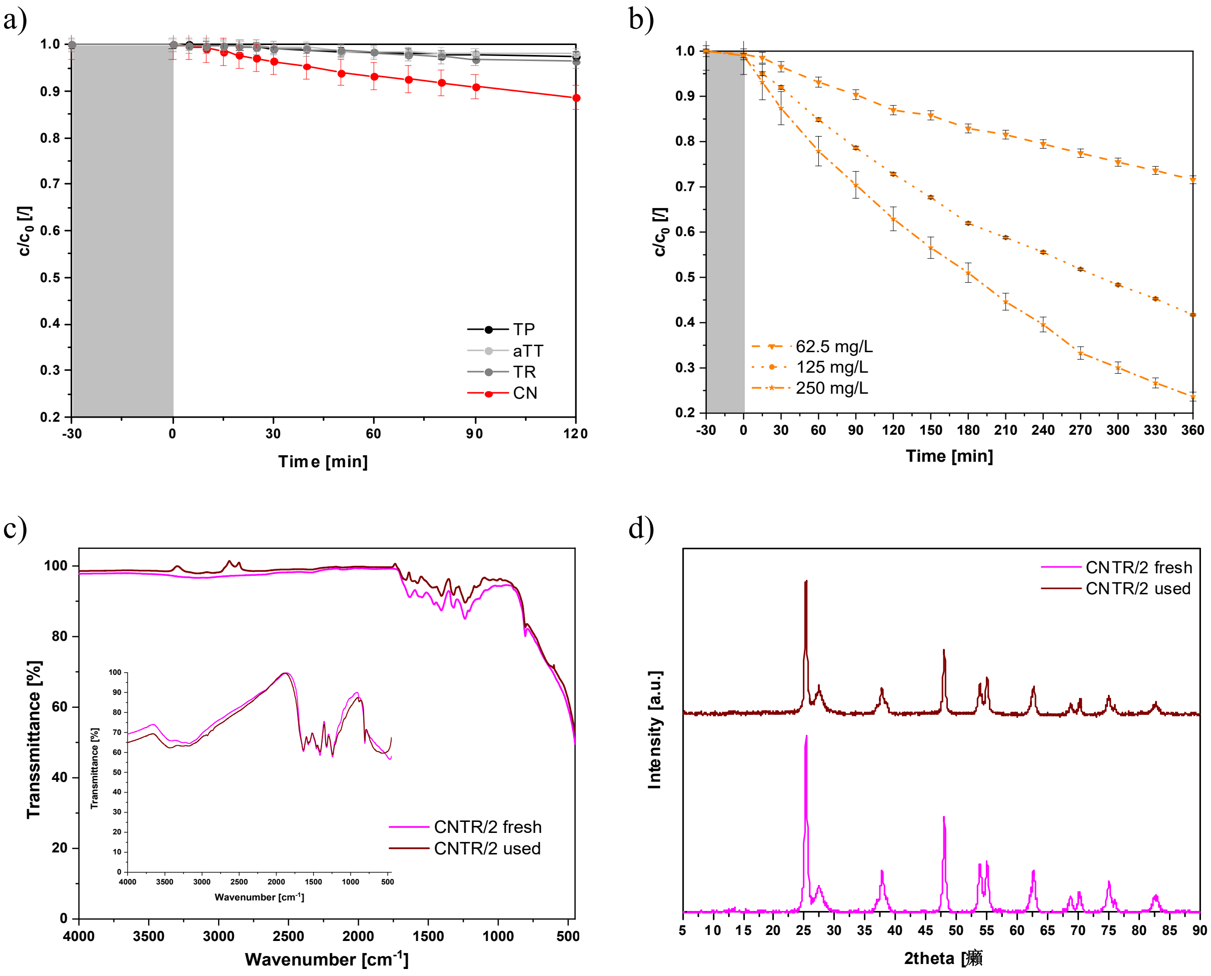

Figure S12. a) Results of BPA degradation with pure components under 2 hours of visible-light irradiation and b) BPA photooxidation with different CNTR/2 concentrations under 6 hours of visible-light. The average ± standard deviation of triplicate tests is shown. Fig. c) shows the ATR-FTIR spectra of fresh and reused CNTR/2 photocatalysts (after 3 BPA degradation cycles), with the inset showing the KBr/CNTR/2 transmission FTIR spectra. Fig. d) shows the XRD patterns of the fresh and reused CNTR/2 samples.

a)

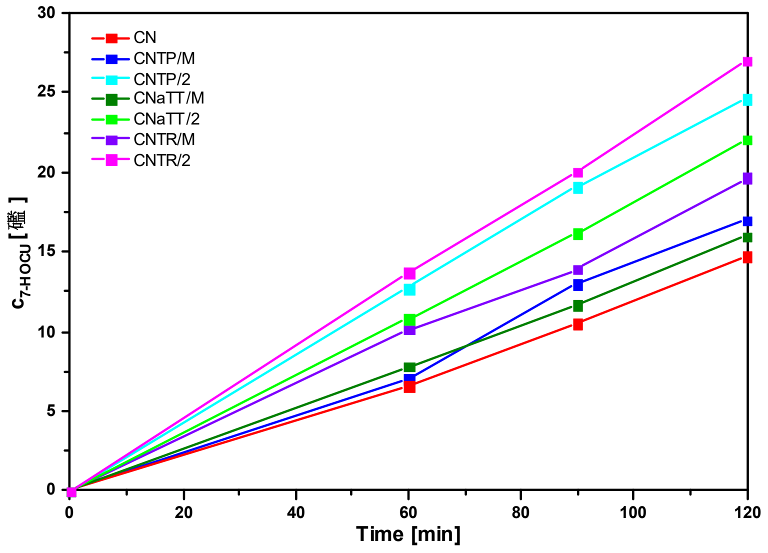

b)

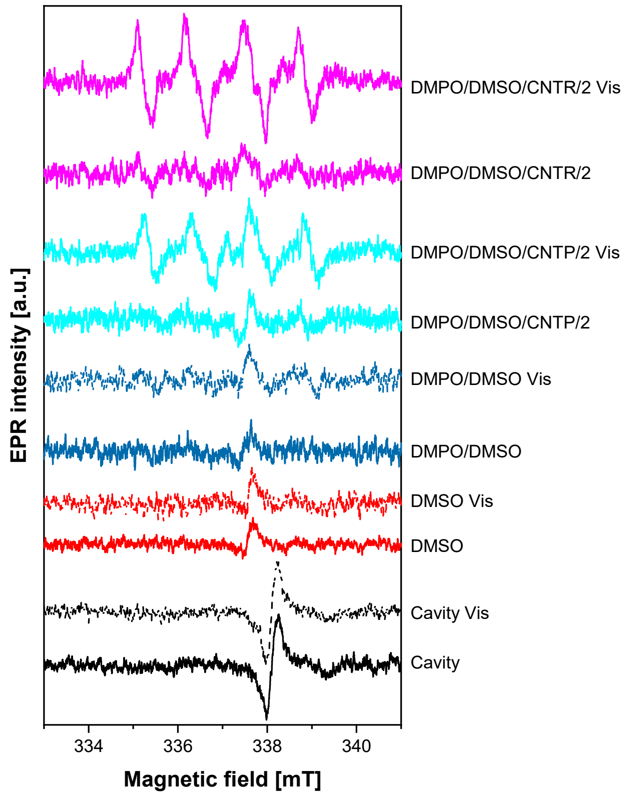

Figure S13. a) Concentration of 7-HOCU after 120 minutes illumination of the catalyst/coumarin suspension with visible-light and b) DMPO/DMSO/catalyst results of the liquid-phase EPR measurements for CNTP/2 and CNTR/2 series after 15 minutes illumination with visible-light.

Table S1

Results of the CHNS elemental analysis of fresh and used photocatalysts. Nitrogen and hydrogen were not detected in the pure TiO_2 samples. Only carbon and hydrogen were measured in the used photocatalysts. The observed error of three repetitions was within ± 1%.

Sample	fresh			used	
	C	N	H	C	H
	wt.%			wt.%	
CN	34.11	61.2	2.0	34.16	1.8
TP	1.41	-	-	1.42	-
CNTP/M	18.72	27.7	0.5	18.81	1.4
CNTP/2	17.59	26.5	0.4	17.71	1.1
aTT	0.04	-	-	0.07	-
CNaTT/M	18.44	28.2	1.5	18.53	1.7
CNaTT/2	17.93	27.0	1.0	18.07	1.2
TR	0.05	-	-	0.06	-
CNTR/M	18.32	27.3	1.0	18.40	1.1
CNTR/2	17.98	26.7	0.9	18.09	1.1

Table S2

Results of the temperature-programmed pyridine desorption measurements of the analysed materials to determine the acidic surface sites (AcSS) (moles of adsorbed pyridine from peak area, calculated concentration and density of AcSS).

Sample	Moles of pyridine	Concentration of AcSS	Density of AcSS
	μmol	$\mu mol/g_{cat}$	$\mu mol/(m^2/g)$
CN	0.9	9.2	0.06
TP	21.9	214.7	0.27
CNTP/M	25.7	256.5	0.45
CNTP/2	18.2	182.4	0.29
aTT	22.1	267.9	0.07
CNaTT/M	31.9	312.1	0.10
CNaTT/2	44.8	453.9	0.27
TR	40.7	428.0	0.41
CNTR/M	31.9	328.5	0.56
CNTR/2	15.7	155.4	0.30

Table S3

Determined point of zero charge (pH_{PZC}, from pH conditioned zeta-potential measurements in Fig. S3), optical band gap value ($^{opt}E_g$, obtained from the UV-Vis DR measurements in Fig. 5) and charge transfer resistance (R_{CT}, obtained from the Nyquist plots in Fig. S7). The observed error of two repetitions for pH_{PZC} was within ± 1% and for R_{CT} within ± 2%. The observed error of three repetitions for $^{opt}E_g$ was within ± 2%.

Sample	pH_{PZC}	$^{opt}E_g$	R_{CT}
	/	eV	MΩ
CN	3.8	2.59	0.83
TP	5.0	3.23	0.96
CNTP/M	5.5	2.70	0.74
CNTP/2	5.1	2.75	0.48
aTT	3.7	3.30	1.0
CNaTT/M	3.7	3.07	0.75
CNaTT/2	4.6	2.99	0.64
TR	5.7	3.19	0.90
CNTR/M	5.3	2.80	0.67
CNTR/2	4.9	2.85	0.40

Table S4

Peak position and peak area of the transitions obtained by the three peak Gaussian deconvolution of the solid-state PL spectra in Fig. S6.

Sample	Peak 1	Peak 2	Peak 3	Cumulative peak position	Area 1	Area 2	Area 3
	nm			nm	a.u.		
CN	447	482	526	462	15060	33504	15781
CNTP/2	435	455	489	443	742	1689	2867
CNaTT/2	437	456	490	450	1995	3970	7829
CNTR/2	442	464	498	453	2170	3497	5954
